# Incorporating Fresh Durum Wheat Semolina Pasta Fortified with Cardoncello (*Pleurotus eryngii*) Mushroom Powder as a Mediterranean Diet Staple

**DOI:** 10.3390/antiox14030284

**Published:** 2025-02-27

**Authors:** Maria Calasso, Alessia Lisi, Arianna Ressa, Giusy Rita Caponio, Graziana Difonzo, Fabio Minervini, Maria Letizia Gargano, Mirco Vacca, Maria De Angelis

**Affiliations:** 1 Department of Soil, Plant and Food Sciences, University of Bari Aldo Moro, Via G. Amendola 165/a, 70126 Bari, Italy; maria.calasso@uniba.it (M.C.); alessia.lisi@uniba.it (A.L.); ariannaressa18@gmail.com (A.R.); graziana.difonzo@uniba.it (G.D.); fabio.minervini@uniba.it (F.M.); marialetizia.gargano@uniba.it (M.L.G.); maria.deangelis@uniba.it (M.D.A.); 2 Department of Bioscience, Biotechnology and Environment, University of Bari “Aldo Moro”, Via Orabona 4, 70125 Bari, Italy; giusy.caponio@uniba.it

**Keywords:** pasta fortification, mushrooms, *Pleurotus eryngii*, high fiber content

## Abstract

Pasta made from durum wheat semolina has a medium–high glycemic index score, high starch digestibility, and limited nutritional value due to its low fiber, vitamin, and bioactive compound content. This study aimed to enhance pasta’s nutritional and functional qualities by incorporating *Pleurotus eryngii* (PE) powder at various substitution levels to achieve one nutritional claim at least. This research involved two phases: evaluating the chemical/physical, nutritional, functional, and sensory properties of laboratory-scale samples and validating the selected formulations through industrial-scale production and shelf-life analyses. The pasta sample with 8.62% PE substitution (SPE8-P) demonstrated significantly improved nutritional qualities, including high fiber content sufficient for a “high fiber content” claim, and potential prebiotic activity indicated by increased bifidobacterial density during simulated fecal microbiota fermentation. Despite its enhanced riboflavin and antioxidant content, regulatory constraints limited the inclusion of claims for vitamin B2 richness and antioxidant activity. Although significantly affecting the color, taste, and odor profiles, the sensory analysis revealed high overall acceptability, supporting the product’s potential for consumer acceptance. This study confirms the feasibility of producing innovative, nutritionally enriched pasta with PE powder as a functional ingredient. Future research will focus on in vivo evaluation to establish the potential for classifying this pasta prototype as a functional food.

## 1. Introduction

Pasta is one of the most consumed staple foods worldwide due to it being a good source of carbohydrates (74–77%), proteins (11–15%), and energy (270–350 kcal/100 g) [1,2]. Durum wheat semolina (*Triticum turgidum* subsp. *durum*) is the exclusive raw material used for the production of pasta thanks to its intense yellow color, high protein content (10.9–13.5%), and ability to provide adequate structural properties in dough [3]. Regarding dry matter, wheat grain is composed of 80–85% floury endosperm, 2–3% germ, and 13–17% bran [4]. Albumins and globulins are the most representative proteins and define a well-balanced amino acid profile [5]. Bran is an excellent source of dietary fiber, rich in minerals, vitamins, and some phytochemicals with antioxidant capacity, such as polyphenols and alkylresorcinols [5,6,7].

According to the classification of foods based on carbohydrates, pasta made with durum wheat semolina is characterized by a medium–high glycemic index score [8], high starch digestibility, and low vitamin, fiber, and bioactive compound content [9]. Therefore, improving the nutritional quality of pasta can be easily achieved by adding ingredients that are able to increase the protein content and/or provide a greater amount of dietary fiber, antioxidants, or other bioactive nutritional components, such as vitamins and minerals. Moreover, the growing understanding and awareness of consumers about the relationship between food and health has led to studies aiming to develop an increased range of functional foods that, taken in adequate quantities, demonstrate an ability to improve the quality of life by preventing nutrition-related diseases [10]. As a consequence, various studies have evaluated the beneficial effects associated with the consumption of pasta produced through the addition of alternative flours or by avoiding the complete refining process [3]. By way of example, previous research has evaluated the effects associated with the consumption of pasta made from buckwheat [11], oats [12], barley [13], sorghum [14], fiber [15], and other agri-food by-products [16,17], with the aim of modifying certain characteristics such as the digestibility of carbohydrates while improving the polyphenolic content—obtaining, in many cases, excellent results.

To avoid the need for very restrictive dietary regimens [18], and in the context of examining ingredients that can be used to produce improved pasta in terms of nutritional quality, *Pleurotus* (*P.*) *eryngii* represents a valid alternative. Commonly called “Cardoncello” in Italy, *P. eryngii* (PE) is a spontaneous mushroom that grows in the Mediterranean area from spring to autumn on the plants of *Eryngium campestrae* and *E. maritimum*. Among the edible mushrooms cultivated, PE is one of the most consumed species in the world [19]. PE is a natural source of bioactive compounds, including beneficial carbohydrates, peptides, dietary fiber, and vitamins, and it exhibits good antioxidant and anti-inflammatory properties [19,20]. Therefore, based on the percentage of substitution and with the aim of developing wheat-based products flagged as healthy, PE represents an effective alternative to increase the total availability of vitamins, minerals, fiber, and β-glucans [21,22,23]. The effect of adding PE in dough was previously studied by researchers, who tested the effect of PE powder or PE fractions in different wheat-based products. In breads, 10% of PE allowed them to obtain a product enriched in vitamins belonging to groups B (thiamin, riboflavin and pantothenic acid) and D (cholecalciferol), as well as total polyphenols and β-glucans [24,25]. In pasta samples, the effect of a 2–6% β-glucan-rich fraction from PE led to a significant increase in dietary fiber and β-glucan content [26]. However, this wheat substitution affects dough’s viscoelastic and rheological properties. As the percentage of wheat replacement increases, the strength of the gluten network decreases, leading to a higher risk of particle loss in unfermented dough and a reduced rising level in leavened bakery products [27]. For this reason, by analyzing the impact of mushroom powder on the microstructure of dough samples, previous authors have suggested 2.5–5.0% PE as the “recommended” percentage of substitution [28]. However, this percentage of substitution does not allow any nutritional claim to be reached.

Therefore, to enhance the nutritional value of fresh semolina pasta while achieving at least one nutritional claim, this research undertook a two-step study. Firstly, the addition of PE or a mixture of whole-wheat semolina and PE at different semolina flour substitution percentages was evaluated, and samples were analyzed to determine differences in terms of chemical/physical composition and nutritional, functional, and sensory aspects. To validate the product, the chosen samples underwent industrial scale-up and chemical/nutritional and shelf-life analyses with the aim of evaluating the differences, while confirming promising outcomes, between samples produced on a laboratory scale and on an industrial level.

## 2. Materials and Methods

### 2.1. Ingredients

Fresh pasta was produced at the laboratory level at the University of Bari Aldo Moro, where dried slices of the “Cardoncello” (*P. eryngii*; PE) mushroom were given by Italmiko S.r.l. (Senise, Italy); both durum wheat semolina (*Triticum durum*; S) and whole-meal semolina (W) were received from Industria Molitoria F.lli Martimucci S.r.l. (Altamura, Italy).

### 2.2. Preparation of Experimental Pasta

In total, 5 kg of PE was ground into a very fine powder using Thermomix TM31 (Vorwerk Contempora Srl, Milan, Italy). The PE powder was stored in a sealed bag at room temperature until use. Together with the related control (CP), 3 experimental doughs containing different percentages of wheat semolina (S) and *P. eryngii* (PE), with or without whole-wheat semolina (W), were produced at the laboratory level, each weighing 500 g (see Table 1).

In detail, doughs were prepared in a mixer (Electrolux assistant, EKM4000, Milan, Italy) by homogenizing the ingredients for 5 min at “low” and 10 min at “high” speeds. These mixtures were rolled by passing them between rollers (Imperia, Italy) to obtain sheets (5 mm thick), which were then cut into the shape of “tagliatelle” (4 mm wide).

### 2.3. pH and TTA of Doughs

The pH of the pasta dough was determined by means of a pH meter (Model 507, Crison, Milan, Italy) with a food penetration probe. The total titratable acidity (TTA) was determined and expressed in a volume (mL) of 0.1 M NaOH solution needed to achieve a pH of 8.3 [29].

### 2.4. Color of Fresh Pasta

The color was measured using a Chromameter CM-600d colorimeter (Konica Minolta Sensing; Osaka, Japan). For each experimental set, 3 different spots from 3 different samples of the same batch were analyzed. The color indices analyzed were brightness (L*), the red index (a*), and the yellow index (b*).

### 2.5. Microbiological Characterization of Pasta

Microbiological analysis of uncooked samples was carried out using aliquots of 25 g homogenized in 225 mL of Buffer Peptone Water 0.1% in a stomacher (Bag Mixer, Interscience International, Roubaix, France) for 2 min [30]. Decimal dilution series of this suspension were prepared and plated onto the following agar media, then incubated according to the regulatory standards. Plate count agar (PCA) (30 °C; 48 h) for total mesophilic aerobic microorganisms, Violet Red Bile Glucose Agar (VRBGA) (37 °C; 24 h) for *Enterobacteriaceae*, Potato Dextrose Agar (PDA) with chloramphenicol (0.1 g/L) (25 °C; 96 h) for molds, Wort agar (WA) with chloramphenicol (0.1 g/L) (25 °C; 48 h) for molds and yeasts, Baird Parker supplemented with Egg Yolk Tellurite (37 °C, 48 h) for staphylococci, and de Man–Rogosa–Sharpe (MRS) agar with cycloheximide (0.1 g/L) (30 °C; 48 h) for LAB were the used media. For LAB, *Enterobacteriaceae*, mold, and yeast counting, the technique of plates by inclusion was used. Additionally, the culturing technique by spreading was performed for staphylococci. The enumeration of molds and yeasts was carried out following microscopic analysis of colony morphology.

### 2.6. Cooking Properties

For each pasta sample, an aliquot (20 g) was cooked in boiling tap water (400 mL) to determine the optimal cooking time (OCT), weight loss during cooking (CL) and Water Absorption Index (WAI) according to the AACC 66-50.01 approved method [31]. In detail, every 30 s of cooking, disappearance of the whitish core of the tagliatelle was evaluated by crushing them between two transparent slides. The moment corresponding to the total disappearance of the nucleus was considered the OCT. To determine weight loss during cooking (CL), i.e., the amount of solid matter lost in the cooking water, three measurements were taken. The Water Absorption Index (WAI) assessed the weight gain of pasta during cooking and was calculated according to the formula below:

$$WAI\left(\%\right)=\frac{weight\;of\;cooked\;pasta-weight\;of\;raw\;pasta}{weight\;of\;raw\;pasta}\times 100$$

### 2.7. Antioxidant Activity

The antioxidant profile was assayed as the scavenging activity against the DPPH∙ free radical and assessing both the total polyphenol (TPC) and total flavonoid (TFC) content. Therefore, 1 g (fresh weight; f.w.) of cooked pasta was added to 10 mL of hydro-alcoholic solution (20:80 *v*/*v*), stirred (VM4 IDL agitator; 10 min, speed 6, at room temperature), treated in an ultrasonic bath for 15 min, and then centrifuged (10,000 rpm, 10 min, 25 °C) [32]. The supernatant of hydroalcoholic extracts was harvested, and pellets underwent a second extraction step following the same procedure. The hydroalcoholic extracts were filtered through a nylon filter (porosity 0.45 mm, Sigma-Aldrich; Burlington, MA, USA).

Antioxidant activity was evaluated in terms of inhibition activity against the radical 2,2-Diphenyl-1-picrilidrazil (DPPH∙), as described by Limongelli et al. [33]. A blank (negative control) and a positive control containing 50 μL of ethanol–water solution or the synthetic antioxidant butylated hydroxytoluene (BHT) were included as a reference (1 g/L in ethanol–water solution), respectively, instead of the sample. Absorbance was measured at 517 nm [34]. In 20 μL of extract, total polyphenols (TPCs) were determined according to the Folin–Ciocalteu test [35]. In the aliquots (1 mL) of each extract, total flavonoids (TFC) were determined by the colorimetric method of aluminum chloride (AlCl_3_) [36]. After incubation (in the dark, 30 min at room temperature), absorbance was measured with a spectrophotometer at 415 nm using an Agilent Cary 60 spectrophotometer (Cernusco, Milan, Italy). Results were derived from a gallic acid calibration curve (0–200 μg/mL) prepared from a stock solution (5 mg/mL in methanol). Results were expressed in mg of gallic acid equivalent (GAE) per mL of extract.

### 2.8. In Vitro Starch Hydrolysis Index

Starch digestion was carried out in vitro according to De Angelis et al. [37]. The starch hydrolysis index (HI) was determined in vitro following the Liljeberg et al.’s method [38]. The weight of white wheat bread (*Triticum aestivum*) containing 1g of starch was used as the reference of the hydrolysis index (HI = 100). Therefore, pasta samples underwent and enzymatic digestion process with simulated oral, gastric, and intestinal fluids. The residual was then subjected to dialysis (membrane porosity 12,400 Da). Intermediate aliquots of dialysate, containing free glucose and partially hydrolyzed starch, were withdrawn from the permeate solution every 30 min and subsequently treated with amyloglucosidase. Hence, free glucose was determined using the D-Fructose/D-Glucose Assay Kit (Megazyme, Wicklow, Ireland) and finally converted to hydrolyzed (digested) starch amounts. The amount of starch digested at 180 min was expressed as the percentage of hydrolyzed starch out of the total determined after 16 h of incubation. The predicted glycemic index (pGI) was calculated using the equation described by Capriles and Arêas [39].

pGI=0.549×HI+39.71

### 2.9. In Vitro Protein Digestibility (IVPD) and Total Free Amino Acid (FAA) Analysis

In 1 g (f.w.) of the sample, the digestibility of proteins in vitro (IVPD) of the cooked samples was determined according to Akeson and Stahmann’s protocol [40], with slight modifications [41]. The concentration of proteins present in the supernatant was determined according to Bradford’s method [42]. The precipitate underwent protein extraction as described by Weiss et al. [43], and the protein concentration was determined. IVPD was expressed as the percentage of solubilized protein fraction after enzymatic hydrolysis compared to the total amount of protein, as expressed with the equation below.

$$IVPD\%=\frac{digested\;amount(peptides+proteins)}{digested\;amount\left(peptides+proteins\right)+undigested\;proteins}\times 100$$

The extract for total free amino acids (FAAs) was prepared from 1 g (f.w.) of the crushed sample added with 4 mL of Tris-HCl 50 mM (pH 8.8) (ratio 1:4 *w*/*v*). As previously detailed [44], samples were left for 1 h at 4 °C to stir and were centrifuged (20 min; 8500× *g*). Both proteins and peptides were precipitated by adding 5% (*w*/*v*) cold solid sulfur–salicylic acid, keeping it at 4 °C for 1h, then centrifuged (10,000× *g*; 15 min; 4 °C). The supernatant was recovered and filtered through a non-sterile 0.22 μm filter and stored at −20 °C until use. From aqueous dough extracts, FAAs were analyzed using the amino acid analyzer Biochrom 30 series (Biochrom Ltd., Cambridge Science Park, UK) with a cation exchange column (inner diameter of 20 × 0.46 cm). A mixture of amino acids at a known concentration was used as reference. FAAs were derivatized post-column with the ninhydrin reagent and detected by absorbance at 440 (proline and hydroxyproline) or 570 nm (all other FAAs).

### 2.10. Simulated Digestion and Colonic Fermentation In Vitro

The digestion of pasta was simulated in vitro considering the enzymatic contribution of the fluids participating in the oral, gastric, and intestinal phases [45]. Aliquots (10 g) of each sample were added to 50 mL of distilled water and mixed in a stomacher (Bag Mixer, Interscience International; Roubaix, France) for 2 min. Subsequently, the solution was mixed with α-amylase (20 mg) in CaCl_2_ (1 mM, 6.25 mL) and incubated at 37 °C for 30 min under stirring conditions to simulate oral digestion. Aiming to simulate gastric digestion conditions, pepsin (2.7 mg) was dissolved in 25 mL of 0.1M HCl, and the mixed sample was added. The pH was raised to values of 2 using HCl 6M and incubated at 37 °C for 3h under shaking conditions. With the aim of simulating the digestion conditions of the small intestine, pancreatin (560 mg) and bile salts (3.5 g/L) were dissolved in 125 mL of 0.1M NaHCO_3_, and the sample was added. The pH was adjusted to 7.0 using NaOH 6M and incubated (37 °C; 3 h) under stirring conditions (50× *g*). At the end of the simulated digestion, the digested paste samples were stored at −20 °C until the next use.

The fecal medium (medium) was prepared as described previously [46]. The supernatant from fecal suspension was supplemented with K_2_HPO_4_•2 g/L, C_2_H_3_NaO_2_•5 g/L, C_6_H_17_N_3_O_7_•2 g/L, MgSO_4_•0.2 g/L, MnSO_4_•0.05 g/L, glucose•2 g/L, inulin•4 g/L, fructo-oligosaccharides•4 g/L, and Tween 80 polysorbate•1 mL/L, then sterilized (121 °C; 20 min). Before proceeding with sterilization, the pH was raised to 7.0 with a NaOH solution (6M). Subsequently, cysteine HCl 0.5 g/L, haemin 0.02 g/L, and vitamin K1 10 μL/L were added. Inulin and fructo-oligosaccharides were supplied by Farmalabor srl, while all other reagents mentioned were supplied by Sigma-Aldrich. The medium was stored at 4 °C. To obtain the fecal microbiota inoculum, 3 fresh fecal samples (<1 h from delivery) from healthy volunteers belonging to the research group were homogenized in distilled water, at 32% (*w*/*v*), with a stomacher (Bag Mixer, Interscience International; Roubaix, France) for 3 min. Recovering the filtered residue, the suspensions were centrifuged (10,000× *g*, 20 min, 4 °C). The supernatant was discarded while the pellets (2 g) were added to the fecal medium suitably complemented with a volume of digest in order to provide 0.5 g of digested pasta. The samples thus prepared were incubated anaerobically at 37 °C under conditions of mild agitation (100× *g*) for 42 h. At the end of the incubation, samples simulating the colonic fermentation phase were used for the count of cultivable microorganisms.

Fecal sample collection was carried out ensuring accordance to the Declaration of Helsinki. The sampling received approval from the Local Ethics Committee (study n. 1572, approved on 5 March 2024), and all subjects signed their informed consent.

### 2.11. Enumeration of Cultivable Fecal Microorganisms

One aliquot (5 g) of each fermented fecal batch was mixed with 45 mL of sterilized saline (0.9% *w*/*v*) and homogenized. According to decimal dilutions, viable bacterial cells were enumerated using the following culture media: Plate count agar (PCA; for total aerobes), Wilkins–Chalgren agar (WC; for total anaerobes), de Man–Rogosa–Sharpe agar (MRS; lactobacilli), M17 agar (streptococci), and Violet-Red Bile Glucose agar (coliforms), *Bifidobacterium* agar (fecal bifidobacteria). Except for *Bifidobacterium* agar (Becton Dickinson; Le Pont de Claix, SA, France), the other media were purchased by Oxoid Ltd. (Basingstoke, Hampshire, UK). Except for WC agar and *Bifidobacterium* agar, the other culture media were incubated aerobically. For the cultivation conditions and times and temperatures used, the instructions provided by the respective manufacturers were followed.

### 2.12. Sensory Analysis

Ensuring accordance with The Code of Ethics of the World Medical Association (Declaration of Helsinki), this study received approval from the Local Ethics Committee (study n. 1572, approved on 5 March 2024), and all subjects signed their informed consent. Sensory analysis was conducted by a panel of ten volunteers (5 F and 5 M) belonging to the research laboratory who were suitably trained on the meaning of sensory attributes and the attribution of scores. Each type of pasta was identified by an alphanumeric code, cooked, and served at room temperature, under natural lighting, in a random and non-repeated order. A complete description of the meaning of each attribute is provided in Appendix A.

### 2.13. Prototype Scale-Up

Based on overall merging and evaluation of the data obtained at the laboratory level concerning the pasta characterization and following an analysis of the management and production costs by the research partner Pastificio Martimucci S.r.l. (Altamura, Italy), we evaluated which sample was suitable for proceeding with the production and packaging of the same at the factory level. Given the choice of dough, a batch of CP and the related prototype of the SPE8 sample were produced, pasteurized, and packaged at the same time as the choice of changing the pasta shape from “tagliatelle” to “trofie”. To produce fortified pasta on a semi-industrial scale, 43.75 kg of semolina was mixed with 6.25 kg of mushroom flour and 10 L of water. The semolina particles had grain sizes in a range of 180–355 μm. Semolina had 15% moisture, ash was 0.86 g/100 g, proteins were 13.3 g/100 g, and dry gluten was 11 g/100 g. The kneading lasted 20 min. The system used (Pavan group, Padua) was Teflon. The moisture yield of the pasta was 30%. The fresh pasta was heat treated. An initial treatment was applied to the bulk product at 73 °C for 3 min. Subsequently, the product packaged in a modified atmosphere (ATM) in 500 g aliquots was re-treated at 73 °C for a further 3 min and stored for 110 days at 4 °C. The same production and thermal process was used for the control pasta. The fresh pasta samples produced were refrigerated at 4 °C until the time of additional analysis.

### 2.14. Prototype Texture Profile Analysis

The texture profile analysis (TPA) was carried out with a FRTS-100N Texture Analyzer (Imada; Toyohashi, Japan) at room temperature equipped with a FR-HA-30J cylindrical probe. For the analysis, pasta samples were cooked until the OCT, left to cool at room temperature, and placed in a beaker (diameter, 100 mm; height 90 mm), filled to about half volume. The selected settings were the following: test speed 1 mm/s, 30% deformation of the sample, and two compression cycles. TPA data were evaluated using Texture Analyzer TVT-XP 3.8.0.5 software (TexVol Instruments; Stockholm, Sweden) [47]. The TPA parameters detected were as follows: hardness (N) (the peak force that occurs during the first compression), resilience (the ability of the pasta to regain its original shape after first compression), fracturability, cohesiveness (calculated as the ratio of the area under the peak of the first bite and area under the peak of the second bite), springiness (cm) (the distance between the beginning and the highest point of the first and the second bites), gumminess (N) (hardness × cohesiveness), and chewiness (N cm) (hardness × cohesiveness × springiness), calculated from the force–time curve [47].

### 2.15. Prototype Shelf Life

To evaluate the shelf life of the SPE8-prototype (thereafter labeled SPE8-P) and related control (PC-P), microbiological analysis was carried out, aimed at enumerating total aerobic mesophilic bacteria (UNI EN ISO 4833-1:2013 method), mesophilic lactic acid bacteria (LAB) (ISO 15214:1998), *Enterobacteriaceae* (ISO 21528-2:2017 method), enterococci (EN ISO 11133:2014), yeasts and molds (ISO 21527-2:2008 standard method), and coagulase-positive staphylococci (UNI EN ISO 6888-2:2004 method). In detail, aliquots of fresh pasta samples (25 g) were aseptically taken from each packet and homogenized in 225 mL of Buffered Peptone Water (BPW) 0.1% in a stomacher (Bag Mixer, Interscience International, Roubaix, France) for 2 min [48]. A series of decimal dilutions of this suspension were prepared and transferred to suitable culture media, in duplicate. The plates were then incubated according to regulatory standards.

### 2.16. Chemical/Nutritional Evaluation of the Prototype

The SPE8-P and PC-P produced, pasteurized, and packaged at Pastificio Martimucci S.r.l. were analyzed for their centesimal composition. Proteins, carbohydrates, vitamins, fiber, fats, ash, and moisture were analyzed using international methods outlined in the table included in Appendix B.

### 2.17. Analysis and Quantification of Phenolic Compounds by UHPLC-DAD-MS/MS

A UHPLC Ultimate 3000RS Dionex interfaced by an H-ESI II probe with an LTQ Velos pro linear ion trap mass spectrometer (Thermo Fisher Scientific, Waltham, MA, USA) was used. The UHPLC system was composed of a quaternary pump, autosampler, column compartment, and detector. As previously reported [49], analytical separation was achieved with some modification; a Hypersil GOLD aQ C18 column (100 mm of length, 2.1 mm internal diameter, and 1.9 μm of particle size) held at 30 °C and at a constant flow of 0.3 mL min^−1^ with water–formic acid (90:10 *v*/*v*) (solvent A) and acetonitrile–formic acid (99.9:0.1 *v*/*v*) (solvent B) was used. The gradient program of solvent A was as follows: 0–20 min from 98% to 30%; 20–24 min isocratic at 30%. Then, equilibration was achieved at the initial conditions for 9 min. The PDA detector was set to scan from 220 to 600 nm of wavelength managed by a 3D field. The MS parameter conditions were taken according to Torreggiani et al. [50], with slight modifications—capillary temperature, 320 °C; source heater temperature, 280 °C; nebulizer gas, N_2_; sheath gas flow, 33 psi; auxiliary gas flow, 5 arbitrary units; and S-Lens RF Level, 60%. Data were acquired in positive ionization mode. Samples were analyzed with two methods: a full scan method from 100 to 1000 *m*/*z* and a data-dependent experiment to collect MS2 data. The data-dependent settings included a full scan from 200 to 1000 for positive ionization, activation level of 500 counts, isolation width of 2 Da, default charge state of 2, and CID energy of 35.

The samples were filtered using syringe filters (LLG Labware, Meckenheim, Germany) in RC by 0.22 µm before injection into the equipment. All data were acquired and processed using Xcalibur v.2 (Thermo Fisher Scientific; MA, USA). The injection volume was 5 μL. A tentative identification of the compounds was performed using mass spectra (MS2), λmax, and retention time in accordance with the literature [51,52]. Quantitative analysis was performed according to the external standard method based on calibration curves obtained by injecting different concentrations of standard solutions (R^2^ = 0.9972). Specifically, the standard used was gallic acid (Sigma-Aldrich; Darmstadt, Germany). Results were expressed in mg of compound per 100 g of sample.

### 2.18. In Vitro Assays of Digested Samples on Cell Cultures

#### 2.18.1. Cell Cultures and Treatment

Caco-2 cells were cultured in Dulbecco’s Modified Eagle Medium (DMEM) GlutaMAX, supplemented with 10% fetal bovine serum (FBS), 100 IU/mL penicillin, 100 μg/mL streptomycin, and 1% non-essential amino acids. Cells were maintained at 37 °C in a humidified atmosphere containing 5% CO_2_ [53]. Cells were either left untreated (basal condition) or treated with digested pasta extracts at two different dilutions (1:200 and 1:100) for 24 h.

#### 2.18.2. Calcein-AM Cell Viability Assay

Cell viability was evaluated using the calcein-AM assay. This assay relies on the conversion of calcein-AM, a non-fluorescent, membrane-permeable compound, into calcein, a green fluorescent, membrane-impermeant compound that accumulates in viable cells with intact plasma membranes. HCT8 cells were treated as previously described [53]. Following treatment, cells were incubated with calcein-AM (1 μM) at 37 °C for 45 min. Fluorescence intensity was subsequently measured using a FLUOstar Omega fluorimeter (BMG LABTECH; Offenburg, Germany). Ethanol (90% for 1 min) served as the internal positive control.

#### 2.18.3. ROS Detection

Intracellular reactive oxygen species (ROS) levels were assessed using dihydrorhodamine-123 (DHR-123). Cells were incubated with DHR-123 (10 μM) at 37 °C for 30 min, after which the medium was replaced with complete DMEM, and cells were further incubated for an additional 30 min. Cells were subsequently lysed using radioimmunoprecipitation assay (RIPA) buffer, composed of 150 mM NaCl, 10 mM Tris-HCl (pH 7.2), 0.1% SDS, 1.0% Triton X-100, 1% sodium deoxycholate, and 5 mM EDTA. Lysates were centrifuged at 12,000× *g* for 10 min at 4 °C, and the resulting supernatants were collected for ROS detection, as previously detailed [32]. For the positive control, cells were treated with tert-butyl hydroperoxide (tBHP, 2 mM) for 30 min. Fluorescence emission was recorded using the FLUOstar Omega fluorimeter (BMG LABTECH), with excitation and emission wavelengths set at 508 nm and 529 nm, respectively.

### 2.19. Statistical Analysis

All analyses were performed in triplicate for each batch of the samples. As indicated by the related caption, data were subjected to a univariate analysis of variance (ANOVA corrected by Tukey’s HSD test) or, alternatively, by Welch’s *t*-test (corrected for multiple comparisons with the Šídák–Bonferroni method). Comparison between the means of the treatments was carried out, with a significance threshold set with values of *p* < 0.05, using the statistical software Statistica 12.5 (StatSoft Inc., Tulsa, OK, USA). Multivariate analysis of principal components (PCA) was carried out on Z-scores by GraphPad Prism v. 9 (GraphPad Software, San Diego, CA, USA).

## 3. Results and Discussion

A substantial portion of the literature has explored how specific foods and diet can sustain health by preventing, slowing the progression of, or treating a wide range of diseases [54,55]. In line with this, the Food and Agriculture Organization (FAO) of the United Nations defines functional foods as “*foods that contain, in addition to nutrients, other components that may be beneficial to health*”. Recently, Temple updated the definition of functional foods as “*novel foods that have been formulated so that they contain substances or live microorganisms that have a possible health-enhancing or disease-preventing value, and at a concentration that is both safe and sufficiently high to achieve the intended benefit. The added ingredients may include nutrients, dietary fiber, phytochemicals, other substances, or probiotics*” [56]. Therefore, both definitions emphasize the necessity of in vivo assessment to confirm beneficial effects. This requirement has restricted the use of the term “functional food” in this research, which instead, as a proof of concept, focuses on the development and evaluation of improved properties in alternative dough for fresh pasta fortified by *P. eryngii* powder compared to conventional dough only containing semolina flour.

### 3.1. Dough pH and Total Titratable Acidity

Samples did not show significant differences for pH values, which ranged between 6.3 ± 0.15 in SPE8 and 6.5 ± 0.4 in CP. Instead, a slight but significant (*p* < 0.05) decrease in TTA values featured SPE17 (1.25 ± 0.35 mL NaOH 0.1N) compared to other samples, of which acidity ranged between 2.1 and 2.5 mL NaOH 0.1N in SPE8 and SWPE, respectively. Thus, the TTA of the samples obtained with the highest percentage of PE inclusion was lower than other samples, despite no differences in pH being observed. This finding could be due to the higher buffering capacity of the significant concentration of proteins in PE powder, resulting from its higher protein content than that of wheat [57]. Indeed, mushrooms are well known for their notable concentration of FAAs, and these influence the sweetness, bitterness, tastelessness, and umami profiles of the resulting products [58], as analyzed in the following subsection.

### 3.2. Colorimetric Analysis of Raw Pasta

**Figure 1:**
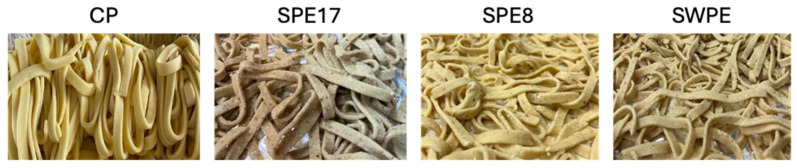
Evaluation of four samples of experimental uncooked pasta. Control sample (CP), samples with different percentages of PE substitution (SPE17 and SPE8), and sample containing both whole-meal semolina and PE (SWPE).

Although previous studies highlighted significant differences between color-related data obtained from colorimeters and those from trained panelists [59], the macroscopic color of pasta is a very important sensory characteristic, markedly influencing consumer decisions [60]. The changes in pasta color after partial replacement of semolina with PE (SPE8, SPE17) or whole-wheat flour and PE (SWPE) are shown analytically (Table 2) and macroscopically (Figure 1). As expected, due to the typical color of the *Pleurotus* mushroom and in line with previous research evaluating differences in colorimetric profiles of PE-enriched products [24,25,26], the addition of PE and whole-wheat flour in the pasta led to a significant decrease (*p* < 0.05) in the brightness value (L*) due to the natural color of PE and whole-wheat flour. The same trend was found for the red index (a*), while the yellow index (b*) followed the opposite trend, with the lowest value detected in PC.

### 3.3. Cultivable Microbiota of Raw Pasta

**Figure 2:**
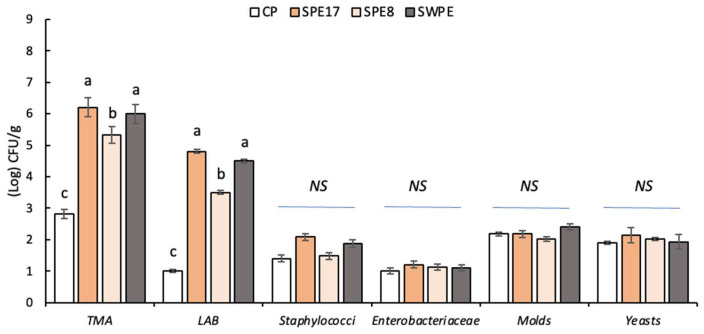
Cell density (Log CFU/g) of cultivable microbiota of the raw pasta. Control sample (CP), samples with different percentages of PE substitution (SPE17 and SPE8), and sample containing both whole-meal semolina and PE (SWPE). Each bar represents the average (±SD) of 3 replicates. On the bars, different letters indicate significant differences (*p* < 0.05; ANOVA corrected by Tukey’s HSD test), while NS (not significant) indicates a *p* > 0.05.

Differences between cultivable microbiota of pasta samples were studied before cooking (Figure 2). Cell densities of total mesophilic aerobes (TMAs) differed significantly between the CP and fortified samples (SPE17, SPE8, SWPE). LAB cell densities almost overlapped those found for TMAs since, as described by Chen et al. [61], the core microbiota of *P. eryngii* hyphae mainly account for the *Lactobacillus* and *Lactococcus* genera. Although *Escherichia-Shigella* species were also reported as prokaryotic cells belonging to the core microbiota of *P. eryngii* [61], the cell densities of *Enterobacteriaceae* showed no significant differences between samples. Similarly, staphylococci, molds, and yeasts were also detected without significant differences between the samples studied.

### 3.4. Cooking Properties

The samples were cooked to determine the OCT and related data on CL and WAI (Table 3). Consistent with previous studies showing comparable OCTs between enriched and non-enriched fresh pasta [62], no significant differences were observed among the samples, with the whitish core disappearing at approximately 10 min on average. A higher percentage of CL was recorded for SPE17 compared to the other samples. This increase in organic substances in the cooking water of SPE17 is likely attributable to the reduced gluten network in the dough, resulting in greater biomass loss [63]. By contrast, WAI values were significantly lower for SWPE than for the other samples. This difference can be attributed to the refining degree of semolina, as refined semolina absorbs more water during cooking than whole-meal semolina due to the presence of more damaged starch [64].

### 3.5. Predictive Glycemic Index

**Figure 3:**
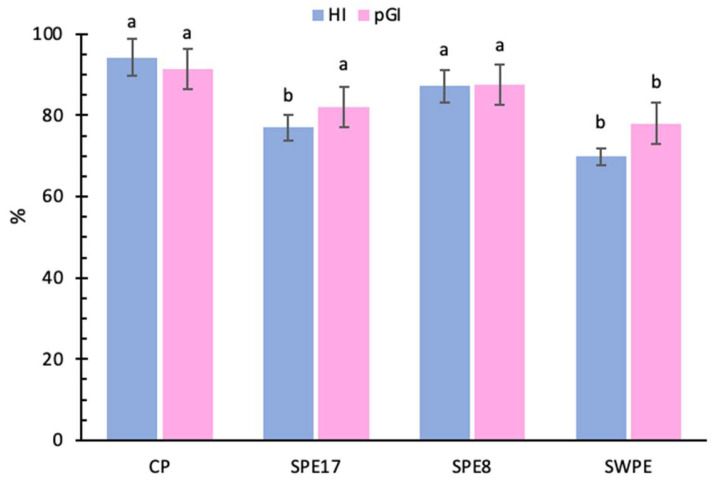
Hydrolysis index (HI) and predictive glycemic index (pGI) percentages of the control sample (CP), samples with different percentages of PE substitution (SPE17 and SPE8), and sample containing both whole-meal semolina and PE (SWPE). Each bar represents the average (±SD) of 3 replicates. On the bars, different letters indicate significant differences (*p* < 0.05; ANOVA corrected by Tukey’s HSD test).

The effect of cooked pasta on glycemia was tested in vitro. CP showed the highest values for both the HI and pGI (Figure 3). These values were significantly higher than those of SWPE. No significant differences were observed between CP and SPE8, while CP and SPE17 differed significantly in HI values but not in pGI. As expected, these differences highlight the impact of substituting wheat with PE, as mushroom-based flours are known to contain fewer glycemia-impacting carbohydrates due to their significant β-glucan content [65,66]. As a soluble fiber, β-glucans play an important role in human health by increasing the viscosity of gastrointestinal contents, which delays gastric emptying [67]. As observed in vivo, this results in reduced nutrient absorption and lower postprandial blood sugar levels [68]. In addition, β-glucans may alter the protein–starch matrix structure, leading to reduced assimilable sugar levels during in vitro digestion [69,70]. Due to the more abundant percentage of refined wheat substitution in SWPE, a significant reduction in pGI value was also observed. This can be attributed to the presence of bran, which has been shown to undergo structural changes during digestion. These changes, such as the loss of surface laminar structures and reduction in particle size, weaken intermolecular forces and contribute to bran’s hypoglycemic effects [71].

### 3.6. In Vitro Protein Digestibility

Since heating leads to structural changes in proteins, protein digestibility was studied in vitro on cooked pasta (Table 4). The assay demonstrated a significantly lower IVPD of SPE17 compared to other samples. Whereas the structure of cooked pasta is generally described as a compact matrix with starch granules trapped in a protein network [72], the lower IVPD of SPE17 could have been caused by the reduced accessibility of enzymes to the network, the lower activity of protease inhibitors, and the lower solubility of proteins [73]. Moreover, previous studies showed that phenolic components were able to form complexes with proteins, leading to a decrease in IVPD [74,75], and, as discussed in the subsection below, SPE17 is featured by the highest total phenol concentration (TPC).

### 3.7. Antioxidant Activity

A diet enriched with bioactive substances can mitigate oxidative stress at the cellular level, helping to prevent complications associated with the accumulation of free radicals and reactive oxygen species (ROS) in the body. Elevated levels of oxidative compounds can cause damage to cell membranes through lipid peroxidation, as well as harm proteins and DNA [76]. Numerous studies have demonstrated a positive correlation between oxidative stress and the onset of neurological and cardiovascular diseases [77].

**Figure 4:**
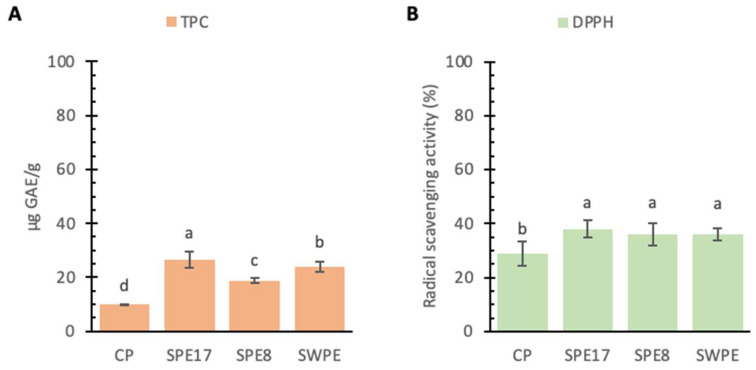
Total phenol content (TPC, panel **A**) and radical scavenging activity (panel **B**) found in the control sample (CP), samples with different percentages of PE substitution (SPE17 and SPE8), and sample containing both whole-meal semolina and PE (SWPE). Each bar represents the average (±SD) of 3 replicates. On the bars, different letters indicate significant differences (*p* < 0.05; ANOVA corrected by Tukey’s HSD test).

Fungi, particularly those of the *Pleurotus* genus, are a rich source of antioxidant polysaccharides, including phenolic compounds, polyketides, terpenes, and steroids [78,79]. Given this evidence, the antioxidant properties of cooked pasta were assessed in terms of TPC and radical scavenging activity (Figure 4). All PE-containing samples exhibited significantly higher TPC values compared to the CP. Among them, SPE17 had the highest concentration (26.43 ± 3.05 μg GAE/g f.w.), while the CP reported the lowest. SWPE demonstrated a significantly higher TPC value compared to SPE8 (23.94 ± 2.19 vs. 18.81 ± 1.79 μg GAE/g f.w.). This increase can be attributed to the phenolic compounds present in the wheat germ and bran of whole-meal semolina [17]. These TPC values were reflected in the radical scavenging activity of the PE-containing samples, all of which showed significantly higher activity than the CP, although no significant differences were observed among the enriched samples themselves. The BHT test further confirmed this trend.

### 3.8. Fecal Microbiota Characterization After In Vitro Simulated Digestion

**Figure 5:**
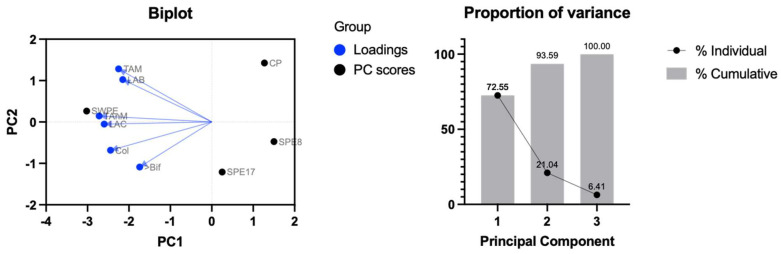
Biplot (loading and PC scores) of the principal component analysis (PCA) of culturable fecal microbiota cell density after simulated (in vitro) colonic fermentation of the control sample (CP), samples with different percentages of PE substitution (SPE17 and SPE8), and sample containing both whole-meal semolina and PE (SWPE). Variable abbreviations: TAMs, total aerobic microbes; TAnM, total anaerobic microbes; LAB, bacillus-shaped lactic acid bacteria; LAC, coccus-shaped lactic acid bacteria; Bif, bifidobacteria; Col, coliforms.

Simulated digestion was conducted in vitro to study the impact of different dough formulations on fecal microbiota. After 42 h of fermentation, plated cell densities were analyzed using a multivariate approach (principal component analysis, PCA). The first two principal components (PC1 and PC2) accounted for 93.59% of the total variability (Figure 5).

Positive scores along PC2 differentiated SWPE and PC samples, while negative scores along PC1 distinctly separated SWPE from the others, indicating a positive influence of SWPE on the overall plated microbial groups. SPE8 and SPE17 demonstrated a similar effect on cultivable microbiota, according to intermediate cell densities of bifidobacteria and coliforms, but reduced densities of total aerobic microbes (TAMs) and lactic acid bacteria (LAB). Although this analysis should be considered preliminary due to limitations in reproducibility related to the use of fecal samples as inoculum, prior studies have highlighted significant effects of wheat and bran fibers on intestinal microbiota. These fibers exhibit prebiotic activity, particularly promoting the growth of bifidobacteria, lactobacilli, and other microbial taxa with saccharolytic metabolism [80,81]. Conversely, the polyphenolic profile of PE, without the substantial prebiotic contribution of bran fibers, may explain the reduced densities of specific microorganisms, particularly TAMs and LAB [82,83].

### 3.9. Sensory Analysis

**Figure 6:**
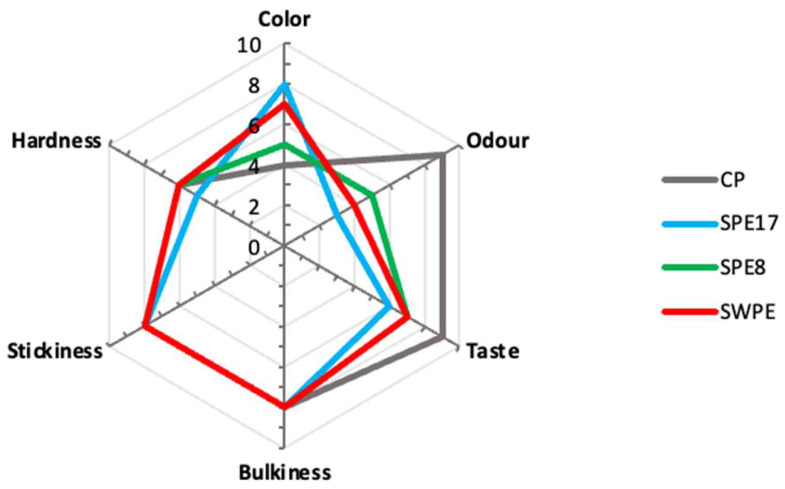
Spider graph of sensory analysis results concerning the control sample (CP), samples with different percentages of PE substitution (SPE17 and SPE8), and sample containing both whole-meal semolina and PE (SWPE).

Concerning the results of the sensory analysis, the addition of PE resulted in fair overall acceptability of the innovative pasta products. However, it should be noted that sensory perception scores of taste and smell attributes were significantly lower for SPE17 compared to the CP (Figure 6). This outcome is likely linked to the high content of volatile aromatic compounds in PE [84] and FAA content [58], which is notably greater than that of other mushroom species within the same genus [85]. It is worth emphasizing that the present sensory evaluation required panelists to compare the innovative pasta to conventional pasta made with durum wheat flour. This framing likely influenced the perception scores for taste and smell. If the evaluation had focused specifically on a “mushroom pasta”, excluding expectations tied to conventional pasta, the perception values might have differed. In line with colorimetric analysis, the color perception scores provided by the panelists were significantly higher (*p* < 0.05) for SPE17 and SWPE samples compared to the CP. This is consistent with the darker appearance of PE-enriched pasta due to its high PE content and, in the case of SWPE, the conspicuous presence of whole-meal semolina. Interestingly, no significant differences were observed in other technological parameters, such as hardness, stickiness, and bulkiness. This suggests that, even with high PE incorporation, these attributes remained unaffected, resulting in a product profile comparable to conventional pasta made solely with durum wheat flour. This observation is consistent with the slight and non-significant differences noted during the evaluation of the cooking properties between samples.

### 3.10. Definition and Characterization of the Prototype

Based on the combined results of the various profiles analyzed, it was evident that a higher percentage of PE incorporation provides significant benefits in terms of increased TPC and reduced pGI. As suggested by previous studies, these improvements may be sufficient to classify the experimental pasta as a fortified food—one with a significantly enhanced nutritional profile compared to conventional pasta [86]. However, these benefits come at the expense of reduced protein digestibility and lower consumer acceptability, particularly regarding taste, smell, hardness, and a stronger perception of color. A similar observation applies to the sample SWPE, where a significant reduction in pGI was assayed but achieved lower consumer acceptability in terms of taste, smell, and color.

Given these findings, the SPE8 sample emerged as the most balanced option. SPE8 demonstrated radical scavenging activity values comparable to those of SPE17 and SWPE while offering greater consumer acceptability during sensory evaluation. Additionally, it maintained a reduced pGI compared to the CP.

**Figure 7:**
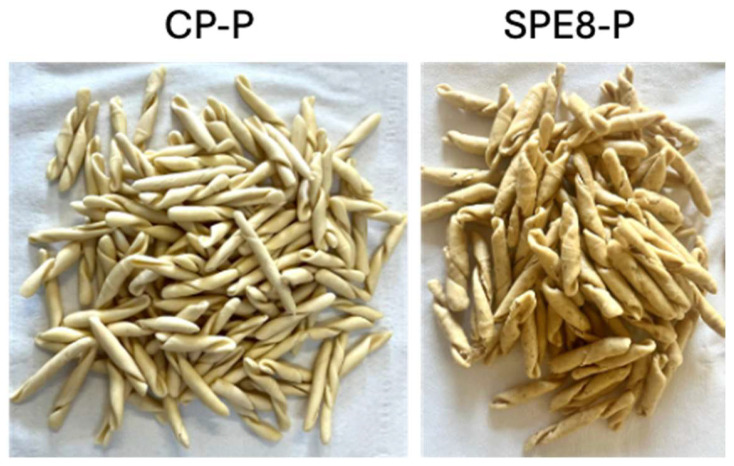
Evaluation of the 2 prototypes of uncooked pasta: the control pasta prototype, CP-P, and the 8.62% PE-containing prototype, SPE8-P.

Therefore, the prototyping process continued with the SPE8 sample. To assess the impact of changing the pasta format from “tagliatelle” to “trofie”, a series of analyses were repeated on the SPE8 prototype (thereafter SPE8-P) and a corresponding CP-P (Figure 7).

### 3.11. Assessment of Differences Between Lab-Produced (Tagliatelle) and Factory-Produced Samples (Trofie)

The assessment focused on comparing lab-produced SPE8 tagliatelle samples with factory-produced trofie, highlighting differences in cooking properties, antioxidant activity, pGI, and nutritional composition.

No significant differences were observed in the degree of acidity, as indicated by pH and TTA measurements.

For cooking properties (Table 5), the change in pasta format from tagliatelle to trofie, although maintaining the same optimal cooking time (OCT) of 10 min, led to noticeable variations in CL (reduced to 5.4%) and WAI (reduced to 38.32%), compared to SPE8, which reported 14.92% and 75.4%, respectively. The CP-P sample followed a similar trend for these parameters.

Regarding brightness, the raw (uncooked) SPE8-P showed mean values of 67.03 (L*), −1.87 (a*), and 23.54 (b*) for brightness, red–green, and yellow–blue indices, respectively (Table 6). In comparison, SPE8 (the lab-made batch) showed values of 62.79 (L*), −0.19 (a*), and 24.02 (b*), revealing differences in brightness and the red index probably due to the pasteurization that occurs in factory-made samples, while the green index remained almost unchanged. These measurements were subsequently repeated in the post-cooked sample, yielding values of 65.88 (L*), −2.67 (a*), and 30.97 (b*). Consistent with previous studies on wheat substitution with PE [24,25,26] these findings confirm that the fortifying ingredient had a more significant impact on brightness and the red–green and yellow–blue indices than the cooking process itself.

The significant decrease in pGI was also assessed in SPE8-P compared to CP-P (79.8 ± 0.9 vs. 82.1 ±0.9, respectively).

Given the pasteurization heat treatment conducted during production at the pasta factory, it was essential to evaluate the antioxidant properties of the SPE8-P and its corresponding CP-P sample. The dual heat treatment significantly impacted the total phenolic content (TPC), reducing it to 0 in CP and 0.001 μg GAE/g. In addition to previous analyses, the total flavonoid content (TFC) was assessed, revealing values of 7.01 ± 0.01 and 17.56 ± 0.55 mg QE/mL for the cooked CP-P and SPE8-P, respectively. Despite the reduction in TPC, the radical scavenging activity assay confirmed a significantly higher antioxidant activity (*p* < 0.05) in the SPE8-P (8.6% ± 0.25) compared to PC-P (4.7% ± 0.12).

The FAA characterization analysis of the cooked prototypes indicated a well-balanced profile of individual amino acids. Notably, most of the amino acids were detected in significantly higher concentrations in the SPE8-P compared to CP-P (Table 7). Previous studies have highlighted how the diverse FAA profiles in mushrooms play a key role in modifying sweet, bitter, tasteless, and umami perceptions [58]. In particular, umami—attributable to the substantial presence of aspartic and glutamic acids—emerged as the characteristic taste of mushrooms, including PE [87].

### 3.12. Texture Profile Analysis

The substitution of semolina with unconventional flour affected the textural attributes of pasta [3]. Texture analysis provides insights into structural parameters closely associated with sensory perceptions [88]. Table 8 presents the texture profile analysis (TPA) of cooked pasta from the control prototype (CP-P) and the 8.62% PE-containing prototype (SPE8-P), evaluated through hardness, cohesiveness, springiness (also referred to as elasticity), gumminess, and chewiness. The primary technological parameters of pasta are typically assessed after cooking, as this process significantly alters the product’s physicochemical properties [89]. Overall, the TPA demonstrated that the addition of PE flour did not significantly affect pasta tenacity, including hardness, resilience, and fracturability. These results likely stem from the absence of significant differences in protein content—and consequently, the protein network—between SPE8-P and CP-P, as well as the strength of the gluten matrix in PE-fortified pasta, which maintained its firmness [90]. The PE-containing prototype exhibited slightly lower cohesiveness values compared to the control sample, a generally reliable indicator of how well the pasta holds together after cooking [91]. Furthermore, the elasticity of SPE8-P pasta was significantly lower (*p* < 0.05) than that of the CP-P control, suggesting a slight weakening of the pasta structure, as evidenced by higher cooking loss (CL) values [91].

### 3.13. Prototype Shelf Life

A shelf-life assessment of the SPE8-P was conducted to evaluate microbiological stability. Following pasteurization, and under MAP, samples were stored at 4 °C and analyzed at intervals of 0, 30, 60, 90, and 110 days.

Throughout the 110-day storage period, the combination of MAP and refrigerated conditions successfully inhibited excessive growth of aerobic microorganisms, maintaining levels within regulatory standards (<10^6^ CFU/g). Both the SPE8-P and the CP-P exhibited similar trends, with total mesophilic aerobes remaining below the threshold for acceptability throughout the storage period. LAB densities remained below 10^1^ CFU/g in both SPE8-P and CP-P, consistent with the absence of significant changes in acidity over time.

Enterobacteria densities remained below 1 Log CFU/g in the CP-P but showed a slight increase in SPE8-P, reaching approximately 1.3 Log CFU/g at 110 days. Molds and yeasts displayed significant increases in SPE8-P at 60 and 90 days, respectively, while the CP-P samples maintained stable cell densities of around 2 Log CFU/g up to 90 days. Presumed coagulase-positive staphylococci were significantly lower in SPE8-P than in CP-P during the entire storage period. Enterococci were not detected in either sample up to 90 days, but at 110 days, SPE8-P exhibited a cell density of approximately 1 Log CFU/g, while no enterococci were observed in the CP-P.

Despite the inclusion of PE, which could potentially impact microbiological stability, the SPE8-P demonstrated a comparable shelf life to the CP-P. These findings underscore the effectiveness of pasteurization and MAP in extending the product’s shelf life under refrigerated conditions. The presence of PE did not compromise the microbiological safety of the SPE8-P, which remained within acceptable standards for up to 110 days.

### 3.14. Chemical/Nutritional Characteristics of the Prototype

The chemical/nutritional analysis of the SPE8-P revealed a significant increase in total fiber content compared to the CP-P (Table 9).

Specifically, SPE8-P showed fiber values of 8.7%, whereas CP contained only 1.5% total fiber. In terms of insoluble fiber, SPE8-P demonstrated values of 5% compared to 0.6% in CP. The increased insoluble fiber content in SPE8-P offers notable benefits to consumers, such as promoting digestive regularity, supporting intestinal health, and aiding in weight management [92]. Additionally, studies suggest that diets rich in fiber, including insoluble fiber, may contribute to reducing the risk of cardiovascular diseases [93]. As well as modulating gut microbiota composition and activity [94], fiber has been shown to directly help in lowering cholesterol levels by binding to cholesterol molecules and facilitating their elimination from the body. It may also play a role in regulating blood sugar levels by slowing the absorption of sugars, thereby maintaining more stable blood sugar levels [95]. Although β-glucans, commonly found in the cell walls of cereals, yeasts, bacteria, and fungi, are well recognized for their roles in cholesterol reduction, blood sugar control, cardiovascular health, immune system modulation, and antioxidant activity, their levels in SPE8 did not meet the threshold required to qualify for an EFSA-recognized health claim [96].

Furthermore, both chemical/nutritional profiles indicated that SPE8-P contained higher levels of vitamin B1 (680 μg/kg) and vitamin B2 (420 μg/kg) compared to CP-P. This is particularly noteworthy since these vitamins cannot be synthesized by the human body and must be obtained through dietary sources.

On the other hand, the analysis found no significant differences in fat or protein content between SPE8-P and CP-P, suggesting that the inclusion of *Pleurotus eryngii* powder did not impact significantly these macronutrient profiles.

### 3.15. Analysis and Quantification of Phenolic Compounds by UHPLC-DAD-MS/MS

**Figure 8:**
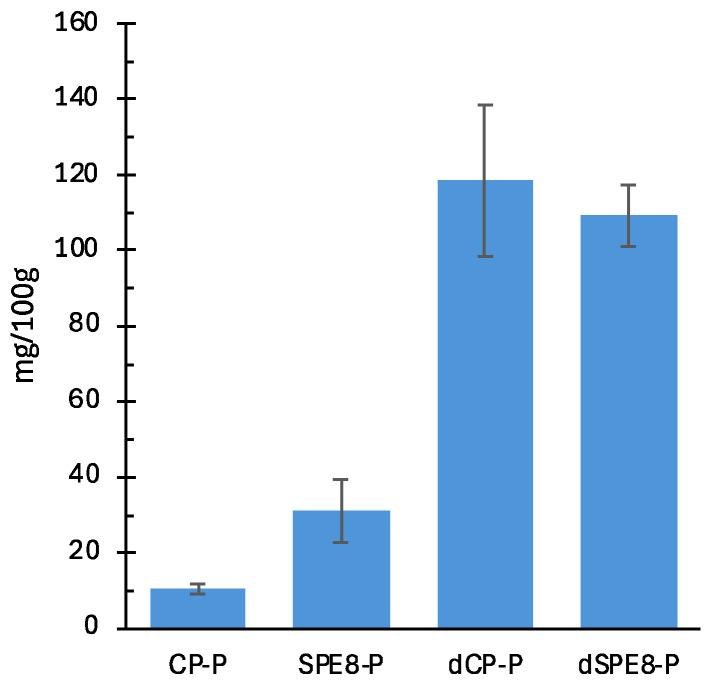
Total phenolic compounds quantified by HPLC-DAD in the control sample (CP-P), samples with PE substitution (SPE-8), and both digested samples (dPC-P, dSPE8-P). Each bar represents the average (±SD) of 2 replicates.

The total concentration of the main compounds detected in each sample and expressed as gallic acid equivalents were assessed (Figure 8). Based on the UV spectra, molecular ions, and the corresponding fragments produced in the MS2 experiment, tentative identification of phenolic compounds was performed. According to the literature [51,52], the main phenolic compounds found in all the samples were presumably phenolic acids. Phenolic acids are present in two forms in cereal grains. Hydroxybenzoic acids are represented by p-hydroxybenzoic, protocatechuic, vanillic, syringic, and gallic acids, while hydroxycinnamic acids include p-coumaric, caffeic, ferulic, and synaptic acids. Ferulic acid is the most prevalent phenolic acid in cereals, representing up to 90% of all phenolic acids [97]. The ferulic acid derivative was identified thanks to the molecular ions and the corresponding fragments produced in the MS2 experiment (*m*/*z* 387, 340, 194, 178, 117) and via comparison with the literature [52]. Ferulic acid is a phenolic compound found in both durum wheat and several species of mushrooms [52,98]. By quantifying the detected phenolic compounds (although a higher concentration of phenolics was detected in the samples containing fungi flour (SPE8-P)), an increase in the TPC, determined by HPLC-DAD, occurred after digestion, regardless of the addition of fungi flour. Consistent with other studies, simulated digestion could increase the total amounts of bio-accessible phenolic compounds [99], with ferulic acid derivatives forming the major bio-accessible phenolic compounds due to their high stability to digestion conditions, as reported previously for barley and wheat sprouts [100].

### 3.16. ROS Detection in Caco-2 Cells Exposed to Digested Prototypes of Pasta

To evaluate the antioxidant activity of the digested pasta extract, Caco-2 cells were treated as previously described, and intracellular ROS content was measured. Initially, cells were exposed to different concentrations of the digested pasta extract (1:200 and 1:100 dilutions) for 24 h to determine the optimal non-cytotoxic concentration using a Calcein-AM cell viability assay. Since neither concentration negatively affected Caco-2 cell viability, the 1:100 dilution was selected for subsequent experiments.

**Figure 9:**
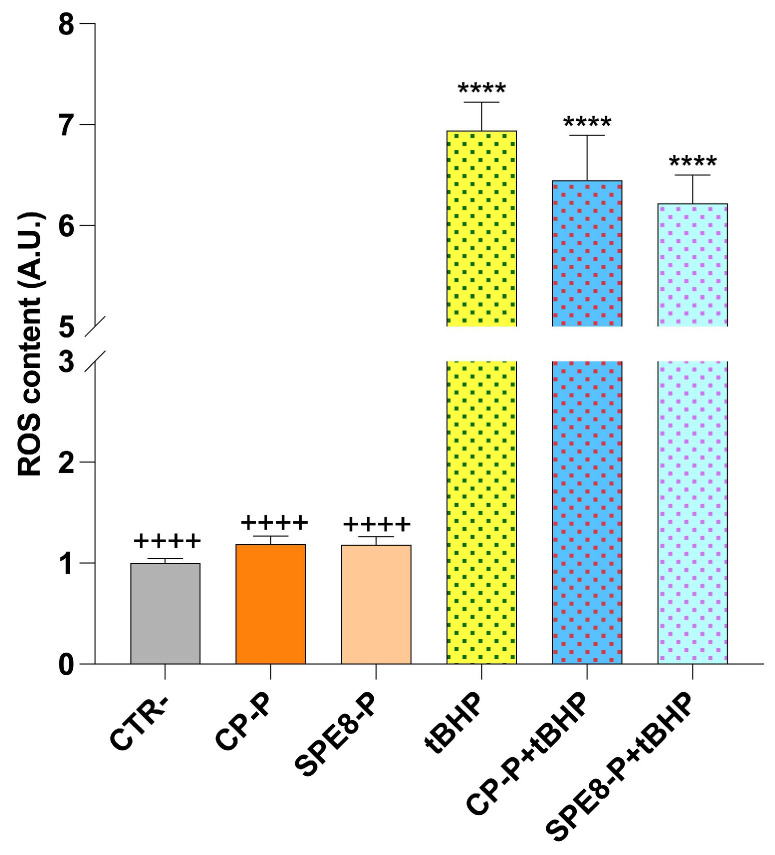
ROS content in Caco-2 cells treated for 24h at a dilution 1:100 of the digested samples. Data are shown as mean ±SD and analyzed by one-way ANOVA followed by Tukey’s multiple comparison test, with (****) showing a significant difference (*p* ≤ 0.05) of samples vs. CTR−, while (++++) shows a significant difference (*p* ≤ 0.05) of samples vs. tBHP.

The digested extracts (CP-P and SPE8-P) did not significantly increase ROS levels in Caco-2 cells (Figure 9), indicating that the extract’s compounds did not exhibit a pro-oxidant effect under basal conditions compared to the control (CTR-). However, when co-incubated with tert-butyl hydroperoxide (tBHP)—a synthetic pro-oxidant compound—the digested pasta extracts (CP-P + tBHP and SPE8-P + tBHP) exhibited a slight, though non-significant, reduction in tBHP-induced intracellular ROS levels, suggesting a potential antioxidant effect.

Notably, this decreasing trend was already evident in the CP-P sample, likely due to the polyphenolic content naturally present in the semolina used in the control formulation [101,102]. Previous studies have shown that mushroom extracts, particularly those derived from *Pleurotus*, contain natural antioxidants capable of reducing intracellular ROS levels and protecting cells from oxidative stress [103,104].

Consistent with this evidence—and supported by the DPPH assay’s results, which demonstrated increased radical scavenging activity in PE pasta samples compared to the control—it is plausible that the percentage of mushroom flour used in the formulation of SPE8-P pasta was insufficient to achieve a statistically significant reduction in intracellular ROS levels in Caco-2 cells.

Therefore, future studies should focus on optimizing pasta formulations with higher concentrations of mushroom flour rich in antioxidants, such as polyphenols and other bioactive compounds, to enhance free radical neutralization and achieve significant ROS level reduction in tumor cells.

## 4. Conclusions

This study confirmed the feasibility of producing pasta enriched with components of high nutritional and functional value by combining semolina with non-conventional ingredients, such as *Pleurotus eryngii*. Using PE at varying substitution levels in fresh pasta significantly enhanced the nutritional profile of this Mediterranean diet staple food. The pasta sample with 8.62% PE substitution (SPE8-P) displayed a high fiber content, sufficient enough to be claimed as pasta with a “high fiber content”. Additionally, analyses in vitro revealed the potential prebiotic activity of SPE8, as evidenced by the significant increase in bifidobacterial cell density during simulated fecal microbiota fermentation. Despite the notable riboflavin content in SPE8-P, it fell short of the 15% recommended daily intake threshold, precluding the opportunity to also be claimed as “rich in vitamin B2”. Similarly, while SPE8 exhibited sufficient antioxidant content to outperform pasta made solely with semolina, the absence of a clearly defined chemical category, as required by EC Regulation, did not allow for the inclusion of a claim related to its high scavenging activity. According to the sensory analysis, which showed the high overall acceptability of the product, we can conclude that this research and development effort—focused on formulating an innovative pasta enhanced with PE for improved nutritional and functional qualities—has significant potential for consumer acceptance. For this reason, the next part of this research will focus on evaluating biological effects in vivo, aiming to evaluate the feasibility of defining this pasta prototype as a functional food.

## Figures and Tables

**Table 1:** Dough composition. CP (control pasta), SPE17 (semolina and 17.24% of PE) ^1^, SPE8 (semolina and 8.62% of PE) ^1^, SWPE (semolina flour, whole-wheat semolina flour, and 8.62% PE). In the table, each value is expressed in grams.

Sample	Semolina	Whole-Wheat Semolina	*P. eryngii* Powder	Water	Dough Weight
ID	(S)	(W)	(PE)		
CP	344.82	-	-	155.17	500
SPE17	258.62	-	86.2	155.17	500
SPE8	301.72	-	43.1	155.17	500
SWPE	77.58	224.13	43.1	155.17	500

^1^ Percentages of PE (17.24% and 8.62%) refer to the final amount of PE powder in doughs.

**Table 2:** Colorimetric analysis of brightness (L*), red–green index (a*), yellow–blue index (b*) of raw pasta. Control sample (CP), samples with different percentages of PE substitution (SPE17 and SPE8), and sample containing both whole-meal semolina and PE (SWPE).

Sample ID	L*	a*	b*
CP	70.86 ± 3.53 a	−4.03 ± −0.23 a	12.47 ± 0.56 b
SPE17	60.39 ± 3.01 b	−0.22 ± −0.04 b	23.36 ± 1.81 a
SPE8	62.79 ± 3.15 b	−0.19 ± −0.07 b	24.02 ± 1.32 a
SWPE	63.73 ± 3.84 b	−0.34 ± −0.09 b	23.77 ± 1.33 a

Values are expressed as mean (±SD) of 3 replicates. Within the same column, different letters indicate a significant difference (*p* < 0.05; ANOVA corrected by Tukey’s HSD test).

**Table 3:** Cooking properties found in control sample (CP), samples with different percentages of PE substitution (SPE17 and SPE8), and sample containing both whole-meal semolina and PE (SWPE).

Sample ID	OCT (min)	CL (%)	WAI (%)
CP	10.0 ± 0.2 a	15.3 ± 0.7 b	83.2 ± 0.4 a
SPE17	9.5 ± 0.6 a	23.8 ± 0.2 a	89.7 ± 0.4 a
SPE8	10.0 ± 0.3 a	14.9 ± 1.1 b	88.9 ± 0.1 a
SWPE	10.2 ± 1.0 a	15.4 ± 0.5 b	75.4 ± 1.1 b

Values are expressed as mean (±SD) of 3 replicates. Within the same column, different letters indicate significant differences (*p* < 0.05; ANOVA corrected by Tukey’s HSD test).

**Table 4:** In vitro protein digestibility (IVPD) of the control sample (CP), samples with different percentages of PE substitution (SPE17 and SPE8), and sample containing both whole-meal semolina and PE (SWPE).

Sample	IVPD (%)
CP	90.08 ± 1.25 a
SPE17	77.21 ± 3.44 b
SPE8	89.24 ± 1.97 a
SWPE	87.57 ± 2.03 a

Values represent the mean (±SD) of 3 replicates. Different letters indicate significant differences (*p* < 0.05; ANOVA corrected by Tukey’s HSD test).

**Table 5:** Cooking properties of control prototype (CP-P) and 8.62% PE-containing prototype (SPE8-P).

Sample ID	OCT (min)	CL (%)	WAI (%)
CP-P	10 ± 0.25 a	3.6 ± 0.1 b	41.0 ± 0.4 a
SPE8-P	10 ± 0.15 a	5.4 ± 0.1 a	38.3 ± 0.1 b

Values are expressed as the mean (±SD) of 3 replicates. Within the same column, different letters indicate significant differences (*p* < 0.05; Welch’s *t*-test corrected for multiple comparisons with the Šídák–Bonferroni method).

**Table 6:** Colorimetric analysis (brightness (L*), red–green index (a*), yellow–blue index (b*)) of uncooked (U) and cooked (C) pasta sample prototypes (CP-P and SPE8-P).

Sample ID	Cooking	L*	a*	b*
CP-P	U	77.73 ± 1.18 a	−7.03 ± 0.15 a	27.82 ± 0.46 a
	C	77.98 ± 2.96 a	−6.32 ± 0.54 a	22.05 ± 0.55 b
SPE8-P	U	67.03 ± 0.38 b	−1.87 ± 0.19 b	23.54 ± 0.23 b
	C	65.88 ± 0.32 b	−2.67 ± 0.50 b	21.98 ± 0.55 b

Values are expressed as the mean (±SD) of 3 replicates. Within the same column, different letters indicate a significant difference (*p* < 0.05; Welch’s *t*-test corrected for multiple comparisons with Šídák–Bonferroni’s method).

**Table 7:** Amino acid composition (mg/kg; ppm) of control prototype (CP-P) and 8.62% PE-containing prototype (SPE8-P).

Amino Acid	CP-P	SPE8-P
Alanine	81.7 ± 2.0	411.7 ± 10.3
Ammonia	20.0 ± 0.5	29.7 ± 0.7
Arginine	47.8 ± 1.2	276.5 ± 6.9
Asparagine	341.4 ± 8.5	258.0 ± 6.4
Aspartic acid	182.4 ± 4.6	148.2 ± 3.7
Cysteine	25.4 ± 0.6	39.5 ± 1.0
Glutamic acid	147.2 ± 3.7	722.3 ± 18.1
Glycine	28.9 ± 0.7	115.6 ± 2.9
Histidine	11.2 ± 0.3	56.3 ± 1.4
Hydroxyproline	-	-
Iso-leucine	36.7 ± 0.9	133.2 ± 3.3
Leucine	25.1 ± 0.6	197.4 ± 4.9
Lysine	22.1 ± 0.6	199.5 ± 5.0
Methionine	6.4 ± 0.2	22.8 ± 0.6
Ornithine	-	368.1 ± 9.2
Phenylalanine	19.1 ± 0.5	104.1 ± 2.6
Proline	-	-
Serine	22.3 ± 0.6	171.0 ± 4.3
Threonine	15.6 ± 0.4	156.2 ± 3.9
Tryptophan	135.7 ± 3.4	107.6 ± 2.7
Tyrosine	19.4 ± 0.5	74.3 ± 1.9
Valine	21.6 ± 0.5	178.4 ± 4.5
γ-aminobutyric acid (GABA)	113.8 ± 2.8	181.0 ± 4.5

Values represent the mean (±SD) of 3 replicates, and all significantly (*p* < 0.05) differed between CP-P and SPE8-P according to the unpaired Welch’s *t*-test corrected for multiple comparisons with Šídák–Bonferroni’s method.

**Table 8:** Textural profile analysis (TPA) of the control pasta prototype (CP-P) and 8.62% PE-containing pasta prototype (SPE8-P).

Textural Features	CP-P	SPE8-P
Hardness (N)	1.41 ± 0.028 a	1.40 ± 0.012 a
Resilience	0.12 ± 0.008	0.11 ± 0.022
Fracturability (N)	0.05 ± 0.02	0.06 ± 0.01
Cohesiveness	0.80 ± 0.015 a	0.74 ± 0.014 b
Springiness (cm)	0.67 ± 0.011 a	0.62 ± 0.012 b
Gumminess (N)	11.30 ± 0.221 a	10.50 ± 0.211b
Chewiness (N cm)	7.55 ± 0.15 a	6.56 ± 0.13 b

Values are expressed as the mean (±SD) of 3 replicates. Within the same column, different letters indicate significant differences (*p* < 0.05; Welch’s *t*-test corrected for multiple comparisons with Šídák–Bonferroni’s method).

**Table 9:** Nutritional facts ^(1)^ of uncooked CP-P and SPE8-P.

Nutritional Facts	CP-P	SPE8-P
Moisture (%)	27.3	24.7
Energy (kcal/100 g)	294	288
Energy (KJ/100 g)	1248	1220
Total carbohydrates (%)	57.6	52.4
Total dietary fiber (%)	1.5	8.7
Insoluble dietary fiber (%)	0.6	5
Soluble dietary fiber (%)	0.9	3.7
(1-3) (1-4) β-glucans (g/100 g)	-	0.85
Total sugars (g/100 g)	1.17	1.74
Glucose (g/100 g)	0.24	0.4
Maltose (g/100 g)	0.7	1.01
Sucrose (g/100 g)	0.23	0.33
Total fats (g/100 g)	1.7	1.6
SFA (%)	0.3	0.3
MUFA (%)	0.35	0.24
PUFA (%)	1.02	1.05
Nitrogen (% N t.q.)	1.82	1.88
Total protein (% N ∗ 6.25 t.q.)	11.4	11.8
Ash (% d.m.)	0.79	1.12
Cd (mg/kg)	0.04	0.05
Ca (mg/kg)	141	116
Mg (mg/kg)	264	260
K (mg/kg)	1406	2887
Na (mg/kg)	2	50
Zn (mg/kg)	6189	7032
Vitamin B1 (thiamine) (µg/kg)	540	680
Vitamin B2 (riboflavin) (µg/kg)	40	622
Salt (g/100 g)	0.001	0.013

^(1)^ The gross composition analysis was carried out by an accredited laboratory according to ISO ISO/IEC 17025 by the Accredia–Italian government authority.

## Data Availability

Raw data concerning the profiling assays are available on request to the corresponding author.

## Abbreviations

The following abbreviations are used in this manuscript:

CL	Weight loss during cooking
CP	Control pasta
CP-P	Control pasta prototype (produced at the factory level)
FAAs	Free amino acids
HI	Starch hydrolysis index
IVPD	In vitro protein digestibility
LAB	Lactic acid bacteria
MAP	Modified atmosphere packaging
MUFA	Monounsaturated fatty acids
OCT	Optimal cooking time
pGI	Predicted glycemic index
PE	*Pleurotus eryngii*
PUFA	Polyunsaturated fatty acids
SFA	Saturated fatty acids
SPE17	Pasta with semolina and 17.24% of PE
SPE8	Pasta with semolina and 8.62% of PE
SPE8-P	Pasta with semolina and 8.62% of PE Prototype (produced at the factory level)
SWPE	Pasta with semolina, 44.82 of whole-wheat semolina flour, and 8.62% PE
TAM	Total aerobes microorganisms
TAnM	Total anaerobes microorganisms
TFC	Total flavonoid content
TMA	Total mesophilic aerobes
TPC	Total phenol content
TTA	Total titratable acidity
WAI	Water Absorption Index

## Appendix A

The sensory analysis attributes considered were color, odor, taste, bulkiness, adhesiveness, hardness, and overall acceptability, using a structured scale from 1 to 10. The typical color of durum wheat pasta was used as a reference index for color, where 1 = yellow color and 10 = brown color. Odor was assessed through smell, and taste was assessed during chewing, with both measured according to the typical smell and taste of durum wheat pasta, where 1 = very low and 10 = very high. The adhesiveness referred to the adhesion index of the strips of tagliatelle pasta to each other, evaluated both visually and manually by pressing two strips of tagliatelle together and determining the force necessary for separation; the adhesiveness was linked to the organic matter released during cooking that still adhered to the surface of the pasta, evaluated by pressing a single thread against the palate and determining the force necessary to remove it with the tongue. Evaluating the hardness involved evaluating the resistance of cooked pasta to chewing, measured by cutting the edge of the tagliatelle with the front teeth.

## Appendix B

For the centesimal composition, the parameters considered were moisture (Method UNI EN ISO 712:2010); energy value (Method Reg. CE 1169/2011GU CE L.304 del 22-11-2011-All. XIV); total carbohydrates (Method L-MI014 rev.0 2016 LOQ: 0.02); total fats (Method DM 23-07-94 SO n°4 G.U. n°186 del 10-08-1994); saturated fatty acids (Method AOAC 996.06); monounsaturated and polyunsaturated fatty acids (method from calculation after the determination of methyl esters according to EEC Reg. 2568/1991 and subsequent amendments); total proteins (Method UNI CEN ISO/TS 16634-2:2016 LOQ: 0.35); total dietary fiber (Method AOAC 985.29 LOQ: 0.1); insoluble and soluble fiber (Method AOAC 993.19:1996 LOQ: 0.1); beta-glucan (Method AOAC 995.16 2005); salt (from calculation after determination of sodium according to L-MI068 LOQ: 0.001); ash (Method ISO 2171:2007); vitamins B1 (Thiamine) (Method L-MI094 rev.0 2022 LOQ: 0.1), B2 (Riboflavin) (Method L-MI094 rev.0 2022 LOQ: 0.1); minerals (calcium, sodium, zinc, magnesium, and potassium) (Met: L-MI068 rev.0 2020LOQ: 10); and heavy metals (cadmium, and lead) (Method L-MI068 rev.0 2020 LOQ: 0.01).

## References

[1] Dziki D. (2021). Current Trends in Enrichment of Wheat Pasta: Quality, Nutritional Value and Antioxidant Properties. Processes.

[2] Melini V., Melini F., Acquistucci R. (2020). Phenolic Compounds and Bioaccessibility Thereof in Functional Pasta. Antioxidants.

[3] Romano A., Ferranti P., Gallo V., Masi P. (2021). New Ingredients and Alternatives to Durum Wheat Semolina for a High Quality Dried Pasta. Curr. Opin. Food Sci..

[4] Durante M., Lenucci M.S., Rescio L., Mita G., Caretto S. (2012). Durum Wheat By-Products as Natural Sources of Valuable Nutrients. Phytochem. Rev..

[5] Catzeddu P., Fois S., Tolu V., Sanna M., Braca A., Vitangeli I., Anedda R., Roggio T. (2023). Quality Evaluation of Fresh Pasta Fortified with Sourdough Containing Wheat Germ and Wholemeal Semolina. Foods.

[6] Hemdane S., Jacobs P.J., Dornez E., Verspreet J., Delcour J.A., Courtin C.M. (2016). Wheat (*Triticum aestivum* L.) Bran in Bread Making: A Critical Review. Comp. Rev. Food Sci. Food Safe.

[7] Pontonio E., Dingeo C., Di Cagno R., Blandino M., Gobbetti M., Rizzello C.G. (2020). Brans from Hull-Less Barley, Emmer and Pigmented Wheat Varieties: From by-Products to Bread Nutritional Improvers Using Selected Lactic Acid Bacteria and Xylanase. Int. J. Food Microbiol..

[8] Lucas-González R., Ángel Pérez-Álvarez J., Moscaritolo S., Fernández-López J., Sacchetti G., Viuda-Martos M. (2021). Evaluation of Polyphenol Bioaccessibility and Kinetic of Starch Digestion of Spaghetti with Persimmon (*Dyospyros kaki*) Flours Coproducts during in Vitro Gastrointestinal Digestion. Food Chem..

[9] Alzuwaid N.T., Pleming D., Fellows C.M., Sissons M. (2021). Fortification of Durum Wheat Spaghetti and Common Wheat Bread with Wheat Bran Protein Concentrate-Impacts on Nutrition and Technological Properties. Food Chem..

[10] Domínguez Díaz L., Fernández-Ruiz V., Cámara M. (2020). An International Regulatory Review of Food Health-Related Claims in Functional Food Products Labeling. J. Funct. Foods.

[11] Vetrani C., Bozzetto L., Giorgini M., Cavagnuolo L., Di Mattia E., Cipriano P., Mangione A., Todisco A., Inghilterra G., Giacco A. (2019). Fibre-Enriched Buckwheat Pasta Modifies Blood Glucose Response Compared to Corn Pasta in Individuals with Type 1 Diabetes and Celiac Disease: Acute Randomized Controlled Trial. Diabetes Res. Clin. Pract..

[12] Liska D.J., Dioum E., Chu Y., Mah E. (2022). Narrative Review on the Effects of Oat and Sprouted Oat Components on Blood Pressure. Nutrients.

[13] Casieri V., Matteucci M., Cavallini C., Torti M., Torelli M., Lionetti V. (2017). Long-Term Intake of Pasta Containing Barley (1–3)Beta-D-Glucan Increases Neovascularization-Mediated Cardioprotection through Endothelial Upregulation of Vascular Endothelial Growth Factor and Parkin. Sci. Rep..

[14] Khan I., Johnson S.K., Yousif A.M., Gamlath S., Ahmad J., Almajwal A.M. (2024). Effect of Sorghum Flour-Containing Pasta on Postprandial Glycemia, Appetite and Energy Intake in Healthy Individuals. Eur. J. Clin. Nutr..

[15] Difonzo G., De Gennaro G., Caponio G.R., Vacca M., Dal Poggetto G., Allegretta I., Immirzi B., Pasqualone A. (2022). Inulin from Globe Artichoke Roots: A Promising Ingredient for the Production of Functional Fresh Pasta. Foods.

[16] Iacobellis I., Lisi A., Vacca M., Apa C.A., Celano G., Mancini L., Minervini F., Calasso M., De Angelis M. (2024). Nutritional, Biochemical, and Functional Properties of Spinach Leaf-Enriched Dough: A Healthier Alternative to Conventional Pasta. Foods.

[17] Vacca M., Khalil M., Rampino A., Celano G., Lanza E., Caponio G.R., Ungaro F., Bertolino A., Di Ciaula A., De Angelis M. (2024). Agreeability and Gastrointestinal Motility Responses to Fully Characterized Experimental Pasta Enriched in Wheat By-Products. J. Funct. Foods.

[18] Celano G., Calabrese F.M., Riezzo G., D’Attoma B., Ignazzi A., Di Chito M., Sila A., De Nucci S., Rinaldi R., Linsalata M. (2024). A Multi-Omics Approach to Disclose Metabolic Pathways Impacting Intestinal Permeability in Obese Patients Undergoing Very Low Calorie Ketogenic Diet. Nutrients.

[19] Biao Y., Chen X., Wang S., Chen G., Mcclements D.J., Zhao L. (2020). Impact of Mushroom (*Pleurotus eryngii*) Flour upon Quality Attributes of Wheat Dough and Functional Cookies-baked Products. Food Sci. Nutr..

[20] Hu Q., Yuan B., Wu X., Du H., Gu M., Han Y., Yang W., Song M., Xiao H. (2019). Dietary Intake of *Pleurotus eryngii* Ameliorated Dextran-Sodium-Sulfate-Induced Colitis in Mice. Mol. Nutr. Food Res..

[21] González A., Cruz M., Losoya C., Nobre C., Loredo A., Rodríguez R., Contreras J., Belmares R. (2020). Edible Mushrooms as a Novel Protein Source for Functional Foods. Food Funct..

[22] Tu J., Brennan M.A., Wu G., Bai W., Cheng P., Tian B., Brennan C.S. (2021). Delivery of Phenolic Compounds, Peptides and β-Glucan to the Gastrointestinal Tract by Incorporating Dietary Fibre-Rich Mushrooms into Sorghum Biscuits. Foods.

[23] Vattapparambil A., Pulickakudy Ajithkumar A., Dubey P.K., Kumar S. (2024). Exploring the Potential of Mushrooms in Ready-to-eat Snack Formulations. Int. J. Food Sci. Technol..

[24] Gaglio R., Guarcello R., Venturella G., Palazzolo E., Francesca N., Moschetti G., Settanni L., Saporita P., Gargano M.L. (2019). Microbiological, Chemical and Sensory Aspects of Bread Supplemented with Different Percentages of the Culinary Mushroom *Pleurotus eryngii* in Powder Form. Int. J. Food Sci. Technol..

[25] Cirlincione F., Venturella G., Gargano M.L., Ferraro V., Gaglio R., Francesca N., Rizzo B.A., Russo G., Moschetti G., Settanni L. (2022). Functional Bread Supplemented with *Pleurotus eryngii* Powder: A Potential New Food for Human Health. Int. J. Gastron. Food Sci..

[26] Kim S., Lee J., Heo Y., Moon B. (2016). Effect of *Pleurotus eryngii* Mushroom β-Glucan on Quality Characteristics of Common Wheat Pasta. J. Food Sci..

[27] Patil S., Sonawane S.K., Mali M., Mhaske S.T., Arya S.S. (2020). Pasting, Viscoelastic and Rheological Characterization of Gluten Free (Cereals, Legume and Underutilized) Flours with Reference to Wheat Flour. J. Food Sci. Technol..

[28] Nie Y., Zhang P., Deng C., Xu L., Yu M., Yang W., Zhao R., Li B. (2019). Effects of *Pleurotus eryngii* (Mushroom) Powder and Soluble Polysaccharide Addition on the Rheological and Microstructural Properties of Dough. Food Sci. Nutr..

[29] Perri G., Minisci A., Montemurro M., Pontonio E., Verni M., Rizzello C.G. (2023). Exploitation of Sprouted Barley Grains and Flour through Sourdough Fermentation. LWT.

[30] Marzano M., Calasso M., Caponio G.R., Celano G., Fosso B., De Palma D., Vacca M., Notario E., Pesole G., De Leo F. (2022). Extension of the Shelf-Life of Fresh Pasta Using Modified Atmosphere Packaging and Bioprotective Cultures. Front. Microbiol..

[31] Padalino L., Mastromatteo M., Lecce L., Spinelli S., Contò F., Del Nobile M.A. (2014). Effect of Durum Wheat Cultivars on Physico-Chemical and Sensory Properties of Spaghetti: Effect of Wheat Cultivars on Spaghetti Properties. J. Sci. Food Agric..

[32] Caponio G.R., Annunziato A., Vacca M., Difonzo G., Celano G., Minervini F., Ranieri M., Valenti G., Tamma G., De Angelis M. (2024). Nutritional, Antioxidant and Biological Activity Characterization of Orange Peel Flour to Produce Nutraceutical Gluten-Free Muffins. Food Funct..

[33] Limongelli R., Minervini F., Calasso M. (2023). Fermentation of Pomegranate Matrices with Hanseniaspora Valbyensis to Produce a Novel Food Ingredient. LWT.

[34] Sharma O.P., Bhat T.K. (2009). DPPH Antioxidant Assay Revisited. Food Chem..

[35] Ainsworth E.A., Gillespie K.M. (2007). Estimation of Total Phenolic Content and Other Oxidation Substrates in Plant Tissues Using Folin–Ciocalteu Reagent. Nat. Protoc..

[36] Chang C.-C., Yang M.-H., Wen H.-M., Chern J.-C. (2002). Estimation of Total Flavonoid Content in Propolis by Two Complementary Colometric Methods. J. Food Drug Anal..

[37] De Angelis M., Rizzello C.G., Alfonsi G., Arnault P., Cappelle S., Di Cagno R., Gobbetti M. (2007). Use of Sourdough Lactobacilli and Oat Fibre to Decrease the Glycaemic Index of White Wheat Bread. Br. J. Nutr..

[38] Liljeberg H., Åkerberg A., Björck I. (1996). Resistant Starch Formation in Bread as Influenced by Choice of Ingredients or Baking Conditions. Food Chem..

[39] Capriles V.D., Arêas J.A.G. (2013). Effects of Prebiotic Inulin-Type Fructans on Structure, Quality, Sensory Acceptance and Glycemic Response of Gluten-Free Breads. Food Funct..

[40] Akeson W.R., Stahmann M.A. (1964). A Pepsin Pancreatin Digest Index of Protein Quality Evaluation. J. Nutr..

[41] Montemurro M., Celano G., De Angelis M., Gobbetti M., Rizzello C.G., Pontonio E. (2020). Selection of Non-Lactobacillus Strains to Be Used as Starters for Sourdough Fermentation. Food Microbiol..

[42] Bradford M.M. (1976). A Rapid and Sensitive Method for the Quantitation of Microgram Quantities of Protein Utilizing the Principle of Protein-Dye Binding. Anal. Biochem..

[43] Weiss W., Vogelmeier C., Görg A. (1993). Electrophoretic Characterization of Wheat Grain Allergens from Different Cultivars Involved in Bakers’ Asthma. Electrophoresis.

[44] Rizzello C.G., Cassone A., Di Cagno R., Gobbetti M. (2008). Synthesis of Angiotensin I-Converting Enzyme (ACE)-Inhibitory Peptides and γ-Aminobutyric Acid (GABA) during Sourdough Fermentation by Selected Lactic Acid Bacteria. J. Agric. Food Chem..

[45] De Angelis M., Siragusa S., Vacca M., Di Cagno R., Cristofori F., Schwarm M., Pelzer S., Flügel M., Speckmann B., Francavilla R. (2021). Selection of Gut-Resistant Bacteria and Construction of Microbial Consortia for Improving Gluten Digestion under Simulated Gastrointestinal Conditions. Nutrients.

[46] Vacca M., Pinto D., Annunziato A., Ressa A., Calasso M., Pontonio E., Celano G., De Angelis M. (2023). Gluten-Free Bread Enriched with Artichoke Leaf Extract In Vitro Exerted Antioxidant and Anti-Inflammatory Properties. Antioxidants.

[47] Rizzello C.G., Verni M., Koivula H., Montemurro M., Seppa L., Kemell M., Katina K., Coda R., Gobbetti M. (2017). Influence of Fermented Faba Bean Flour on the Nutritional, Technological and Sensory Quality of Fortified Pasta. Food Funct..

[48] Calasso M., Marzano M., Caponio G.R., Celano G., Fosso B., Calabrese F.M., De Palma D., Vacca M., Notario E., Pesole G. (2023). Shelf-Life Extension of Leavened Bakery Products by Using Bio-Protective Cultures and Type-III Sourdough. LWT.

[49] Difonzo G., Troilo M., Allegretta I., Pasqualone A., Caponio F. (2023). Grape Skin and Seed Flours as Functional Ingredients of Pizza: Potential and Drawbacks Related to Nutritional, Physicochemical and Sensory Attributes. LWT.

[50] Torreggiani A., Demarinis C., Pinto D., Papale A., Difonzo G., Caponio F., Pontonio E., Verni M., Rizzello C.G. (2023). Up-Cycling Grape Pomace through Sourdough Fermentation: Characterization of Phenolic Compounds, Antioxidant Activity, and Anti-Inflammatory Potential. Antioxidants.

[51] Nicoletti I., Martini D., De Rossi A., Taddei F., D’Egidio M.G., Corradini D. (2013). Identification and Quantification of Soluble Free, Soluble Conjugated, and Insoluble Bound Phenolic Acids in Durum Wheat (*Triticum turgidum* L. Var. durum) and Derived Products by RP-HPLC on a Semimicro Separation Scale. J. Agric. Food Chem..

[52] Yahia E.M., Gutiérrez-Orozco F., Moreno-Pérez M.A. (2017). Identification of Phenolic Compounds by Liquid Chromatography-Mass Spectrometry in Seventeen Species of Wild Mushrooms in Central Mexico and Determination of Their Antioxidant Activity and Bioactive Compounds. Food Chem..

[53] Khalil M., Piccapane F., Vacca M., Celano G., Mahdi L., Perniola V., Apa C.A., Annunziato A., Iacobellis I., Procino G. (2024). Nutritional and Physiological Properties of Thymbra Spicata: In Vitro Study Using Fecal Fermentation and Intestinal Integrity Models. Nutrients.

[54] Clark M., Hill J., Tilman D. (2018). The Diet, Health, and Environment Trilemma. Annu. Rev. Environ. Resour..

[55] Zmora N., Suez J., Elinav E. (2019). You Are What You Eat: Diet, Health and the Gut Microbiota. Nat. Rev. Gastroenterol. Hepatol..

[56] Temple N.J. (2022). A Rational Definition for Functional Foods: A Perspective. Front. Nutr..

[57] Mennah-Govela Y.A., Singh R.P., Bornhorst G.M. (2019). Buffering Capacity of Protein-Based Model Food Systems in the Context of Gastric Digestion. Food Funct..

[58] Lavelli V., Proserpio C., Gallotti F., Laureati M., Pagliarini E. (2018). Circular Reuse of Bio-Resources: The Role of *Pleurotus* Spp. in the Development of Functional Foods. Food Funct..

[59] Milovanović B., Đekić I., Sołowiej B., Novaković S., Đorđevic V., Tomašević I. (2020). Computer Vision System: A Better Tool for Assessing Pork and Beef Colour than a Standard Colourimeter. Meat Technol..

[60] Bianchi F., Tolve R., Rainero G., Bordiga M., Brennan C.S., Simonato B. (2021). Technological, Nutritional and Sensory Properties of Pasta Fortified with Agro-industrial By-products: A Review. Int. J. Food Sci. Technol..

[61] Chen L., Yan M., Qian X., Yang Z., Xu Y., Wang T., Cao J., Sun S. (2022). Bacterial Community Composition in the Growth Process of *Pleurotus eryngii* and Growth-Promoting Abilities of Isolated Bacteria. Front. Microbiol..

[62] Drabińska N., Nogueira M., Ciska E., Jeleń H. (2022). Effect of Drying and Broccoli Leaves Incorporation on the Nutritional Quality of Durum Wheat Pasta. Pol. J. Food Nutr. Sci..

[63] Ungureanu-Iuga M., Dimian M., Mironeasa S. (2020). Development and Quality Evaluation of Gluten-Free Pasta with Grape Peels and Whey Powders. LWT.

[64] Sissons M., Abecassis J., Marchylo B., Carcea M. (2012). Durum Wheat: Chemistry and Technology.

[65] Carbonero E.R., Gracher A.H.P., Smiderle F.R., Rosado F.R., Sassaki G.L., Gorin P.A.J., Iacomini M. (2006). A β-Glucan from the Fruit Bodies of Edible Mushrooms *Pleurotus eryngii* and *Pleurotus ostreatoroseus*. Carbohydr. Polym..

[66] Synytsya A., Míčková K., Synytsya A., Jablonský I., Spěváček J., Erban V., Kováříková E., Čopíková J. (2009). Glucans from Fruit Bodies of Cultivated Mushrooms *Pleurotus ostreatus* and *Pleurotus eryngii*: Structure and Potential Prebiotic Activity. Carbohydr. Polym..

[67] Foschia M., Peressini D., Sensidoni A., Brennan M.A., Brennan C.S. (2015). Synergistic Effect of Different Dietary Fibres in Pasta on in Vitro Starch Digestion?. Food Chem..

[68] Bozbulut R., Şanlıer N., Döğer E., Bideci A., Çamurdan O., Cinaz P. (2020). The Effect of Beta-Glucan Supplementation on Glycemic Control and Variability in Adolescents with Type 1 Diabetes Mellitus. Diabetes Res. Clin. Pract..

[69] Yang Y., Wang Y., Zhang R., Jiao A., Jin Z. (2024). The Impact of Different Soluble Endogenous Proteins and Their Combinations with β-Glucan on the in Vitro Digestibility, Microstructure, and Physicochemical Properties of Highland Barley Starch. Int. J. Biol. Macromol..

[70] Shi M., Song X., Chen J., Ji X., Yan Y. (2024). Effect of Oat Beta-Glucan on Physicochemical Properties and Digestibility of Fava Bean Starch. Foods.

[71] Neylon E., Arendt E.K., Zannini E., Sahin A.W. (2021). Fundamental Study of the Application of Brewers Spent Grain and Fermented Brewers Spent Grain on the Quality of Pasta. Food Struct..

[72] Petitot M., Abecassis J., Micard V. (2009). Structuring of Pasta Components during Processing: Impact on Starch and Protein Digestibility and Allergenicity. Trends Food Sci. Technol..

[73] Kamble D.B., Singh R., Rani S., Kaur B.P., Upadhyay A., Kumar N. (2019). Optimization and Characterization of Antioxidant Potential, in Vitro Protein Digestion and Structural Attributes of Microwave Processed Multigrain Pasta. J. Food Process Preserv..

[74] Ozdal T., Capanoglu E., Altay F. (2013). A Review on Protein–Phenolic Interactions and Associated Changes. Food Res. Int..

[75] Sęczyk Ł., Gawlik-Dziki U., Świeca M. (2021). Influence of Phenolic-Food Matrix Interactions on In Vitro Bioaccessibility of Selected Phenolic Compounds and Nutrients Digestibility in Fortified White Bean Paste. Antioxidants.

[76] Zheng Y., Sun J., Luo Z., Li Y., Huang Y. (2024). Emerging Mechanisms of Lipid Peroxidation in Regulated Cell Death and Its Physiological Implications. Cell Death Dis..

[77] Hassan W., Noreen H., Rehman S., Kamal M.A., Da Rocha J.B.T. (2022). Association of Oxidative Stress with Neurological Disorders. Curr. Neuropharmacol..

[78] Chun S., Gopal J., Muthu M. (2021). Antioxidant Activity of Mushroom Extracts/Polysaccharides—Their Antiviral Properties and Plausible AntiCOVID-19 Properties. Antioxidants.

[79] Petraglia T., Latronico T., Fanigliulo A., Crescenzi A., Liuzzi G.M., Rossano R. (2023). Antioxidant Activity of Polysaccharides from the Edible Mushroom *Pleurotus eryngii*. Molecules.

[80] D’hoe K., Conterno L., Fava F., Falony G., Vieira-Silva S., Vermeiren J., Tuohy K., Raes J. (2018). Prebiotic Wheat Bran Fractions Induce Specific Microbiota Changes. Front. Microbiol..

[81] Jefferson A., Adolphus K. (2019). The Effects of Intact Cereal Grain Fibers, Including Wheat Bran on the Gut Microbiota Composition of Healthy Adults: A Systematic Review. Front. Nutr..

[82] Piekarska-Radzik L., Klewicka E. (2021). Mutual Influence of Polyphenols and *Lactobacillus* spp. Bacteria in Food: A Review. Eur. Food Res. Technol..

[83] Makarewicz M., Drożdż I., Tarko T., Duda-Chodak A. (2021). The Interactions between Polyphenols and Microorganisms, Especially Gut Microbiota. Antioxidants.

[84] Zhu M., Hu Z., Liang M., Song L., Wu W., Li R., Li Z., Zhang J. (2022). Evaluation of the Flavor Compounds of *Pleurotus eryngii* as Affected by Baking Temperatures Using HS-SPME-GC–MS and Electronic Nose. Food Process. Preserv..

[85] Tagkouli D., Bekiaris G., Pantazi S., Anastasopoulou M.E., Koutrotsios G., Mallouchos A., Zervakis G.I., Kalogeropoulos N. (2021). Volatile Profiling of *Pleurotus eryngii* and Pleurotus Ostreatus Mushrooms Cultivated on Agricultural and Agro-Industrial By-Products. Foods.

[86] Pontonio E., Montemurro M., Dingeo C., Rotolo M., Centrone D., Carofiglio V.E., Rizzello C.G. (2022). Design and Characterization of a Plant-Based Ice Cream Obtained from a Cereal/Legume Yogurt-Like. LWT.

[87] Kurihara K. (2009). Glutamate: From Discovery as a Food Flavor to Role as a Basic Taste (Umami). Am. J. Clin. Nutr..

[88] Montemurro M., Coda R., Rizzello C. (2019). Recent Advances in the Use of Sourdough Biotechnology in Pasta Making. Foods.

[89] Rosa-Sibakov N., Heiniö R.-L., Cassan D., Holopainen-Mantila U., Micard V., Lantto R., Sozer N. (2016). Effect of Bioprocessing and Fractionation on the Structural, Textural and Sensory Properties of Gluten-Free Faba Bean Pasta. LWT-Food Sci. Technol..

[90] El-Sohaimy S.A., Brennan M., Darwish A.M.G., Brennan C. (2020). Physicochemical, Texture and Sensorial Evaluation of Pasta Enriched with Chickpea Flour and Protein Isolate. Ann. Agric. Sci..

[91] Flores-Silva P.C., Berrios J.D.J., Pan J., Agama-Acevedo E., Monsalve-González A., Bello-Pérez L.A. (2015). Gluten-Free Spaghetti with Unripe Plantain, Chickpea and Maize: Physicochemical, Texture and Sensory Properties. CyTA J. Food.

[92] Baky M.H., Salah M., Ezzelarab N., Shao P., Elshahed M.S., Farag M.A. (2024). Insoluble Dietary Fibers: Structure, Metabolism, Interactions with Human Microbiome, and Role in Gut Homeostasis. Crit. Rev. Food Sci. Nutr..

[93] Zhang L., Chen Y., Yang Q., Guo J., Zhou S., Zhong T., Xiao Y., Yu X., Feng K., Peng Y. (2025). The Impact of Dietary Fiber on Cardiovascular Diseases: A Scoping Review. Nutrients.

[94] De Angelis M., Garruti G., Minervini F., Bonfrate L., Portincasa P., Gobbetti M. (2019). The Food-Gut Human Axis: The Effects of Diet on Gut Microbiota and Metabolome. Curr. Med. Chem..

[95] Islam S.U., Ahmed M.B., Ahsan H., Lee Y.-S. (2021). Recent Molecular Mechanisms and Beneficial Effects of Phytochemicals and Plant-Based Whole Foods in Reducing LDL-C and Preventing Cardiovascular Disease. Antioxidants.

[96] Harland J. (2014). Authorised EU Health Claims for Barley and Oat Beta-Glucans. Foods, Nutrients and Food Ingredients with Authorised EU Health Claims.

[97] Dvořáček V., Jágr M., Jelínek M., Polišenská I., Spitzer T., Hermuth J. (2024). Phenolic Acids and Their Relationship to Nutritional and Technological Grain Parameters of Durum Wheat Under Variable Treatment Intensity in Central European Conditions. Agronomy.

[98] Çayan F., Deveci E., Tel-Çayan G., Duru M.E. (2020). Identification and Quantification of Phenolic Acid Compounds of Twenty-Six Mushrooms by HPLC–DAD. Food Meas..

[99] Tomé-Sánchez I., Martín-Diana A.B., Peñas E., Frias J., Rico D., Jiménez-Pulido I., Martínez-Villaluenga C. (2021). Bioprocessed Wheat Ingredients: Characterization, Bioaccessibility of Phenolic Compounds, and Bioactivity During in Vitro Digestion. Front. Plant Sci..

[100] Aborus N.E., Šaponjac V.T., Čanadanović-Brunet J., Ćetković G., Hidalgo A., Vulić J., Šeregelj V. (2018). Sprouted and Freeze-Dried Wheat and Oat Seeds—Phytochemical Profile and in Vitro Biological Activities. Chem. Biodivers..

[101] Kamble D.B., Singh R., Pal Kaur B., Rani S., Upadhyay A. (2020). Effect of Microwave Processing on Physicothermal Properties, Antioxidant Potential, in Vitro Protein Digestibility and Microstructure of Durum Wheat Semolina. Food Meas..

[102] Liyana-Pathirana C.M., Shahidi F. (2006). Importance of Insoluble-Bound Phenolics to Antioxidant Properties of Wheat. J. Agric. Food Chem..

[103] Khan A.A., Gani A., Masoodi F.A., Khanday F.A. (2020). Antioxidant, Antiproliferative, Immunomodulatory, Antimicrobial and Functional Properties of Wild Mushroom (*Coprinus atramentarius*) β-Glucan Extract as Affected by γ-Irradiation Treatment. Can. J. Clin. Nutr..

[104] Arunachalam K., Sreeja P.S., Yang X. (2022). The Antioxidant Properties of Mushroom Polysaccharides Can Potentially Mitigate Oxidative Stress, Beta-Cell Dysfunction and Insulin Resistance. Front. Pharmacol..

